# The Tail Wags the Dog: The Far Periphery of the Coordination Environment Manipulates the Photophysical Properties of Heteroleptic Cu(I) Complexes

**DOI:** 10.3390/molecules27072250

**Published:** 2022-03-30

**Authors:** Aleksandra Paderina, Alexey Melnikov, Sofia Slavova, Vladimir Sizov, Vladislav Gurzhiy, Stanislav Petrovskii, Maksim Luginin, Oleg Levin, Igor Koshevoy, Elena Grachova

**Affiliations:** 1 Institute of Chemistry, St. Petersburg University, Universitetskii pr. 26, 198504 St. Petersburg, Russia; ksaniasha@list.ru (A.P.); sizovvv@mail.ru (V.S.); s.petrovsky@spbu.ru (S.P.); maclugin@yandex.ru (M.L.); o.levin@spbu.ru (O.L.); 2 Centre for Nano- and Biotechnologies, Peter the Great St. Petersburg Polytechnic University, 195251 St. Petersburg, Russia; amelnikov-noc@yandex.ru; 3 Institute of General and Inorganic Chemistry, Bulgarian Academy of Sciences, 1113 Sofia, Bulgaria; sslavova@svr.igic.bas.bg; 4 Institute of Earth Sciences, St. Petersburg University, 199034 St. Petersburg, Russia; vladgeo17@mail.ru; 5 Department of Chemistry, University of Eastern Finland, 80101 Joensuu, Finland; igor.koshevoy@uef.fi

**Keywords:** copper(I) complex, bipyridine derivative, luminescence, quantum chemical calculation

## Abstract

In this work we show, using the example of a series of [Cu(Xantphos)(N^N)]^+^ complexes (N^N being substituted 5-phenyl-bipyridine) with different peripheral N^N ligands, that substituents distant from the main action zone can have a significant effect on the physicochemical properties of the system. By using the C≡C bond on the periphery of the coordination environment, three hybrid molecular systems with −Si(CH_3_)_3_, −Au(PR_3_), and −C_2_HN_3_(CH_2_)C_10_H_7_ fragments were produced. The Cu(I) complexes thus obtained demonstrate complicated emission behaviour, which was investigated by spectroscopic, electrochemical, and computational methods in order to understand the mechanism of energy transfer. It was found that the −Si(CH_3_)_3_ fragment connected to the peripheral C≡C bond changes luminescence to long-lived intra-ligand phosphorescence, in contrast to MLCT phosphorescence or TADF. The obtained results can be used for the design of new materials based on Cu(I) complexes with controlled optoelectronic properties on the molecular level, as well as for the production of hybrid systems.

## 1. Introduction

Molecular emitters based on metal complexes demonstrate luminescence in the visible and NIR range in response to photoexcitation by UV or visible light. These kinds of systems have potential wide-range applications in various modern technologies such as sensing [1,2], production of optoelectronic devices [3], photocatalysis [4], theranostics [5], photovoltaics [6], etc. The appropriate design of molecular emitters mainly determines its future physicochemical characteristics. For instance, molecular emitters contain a transition metal and connect directly to an organic chromophore moiety, leading to different luminescence mechanisms [7,8]. On the other hand, the photophysical behaviour of the complex depends on the nature of the metal centre, mostly on the synergy of properties between the metal centre and the coordination environment. However, due to the non-additive contribution of each component in photochemistry of molecular emitters, the identification of relationships between the metal centre and the coordination environment and the subsequent prediction of ‘composition/property’ correlation remains a complicated issue.

It should be mentioned that photophysical properties can play the role of an indicator, reflecting changes in the electronic structure of the metal–ligand system and allowing the monitoring of the relationship between ligand composition and the physicochemical properties of the complex. From this perspective, the formation of a complex with desired photophysical properties can be realized by modifying the composition of the coordination sphere close to the metal centre. It is assumed that any changes at the distant periphery of the ligand environment do not significantly adjust the properties of the system as a whole. This is a universal strategy for molecular emitter design, and is widely used for creating ‘hybrid’ complex systems. For example, the approach mentioned above has been favourably applied to systems with covalent conjugations of a polymer and a complex, where it is important to retain the original physicochemical properties of the initial complex [9,10,11].

Cu(I) complexes provide an excellent testing ground for studying mutual impacts between ligand composition and compound properties by monitoring of photoluminescence. In this light, copper is an attractive alternative to precious metals both with regard to cost and environmental safety [12,13,14,15]. Another proficient reason to study Cu(I) molecular emitters is their ability to exhibit thermally activated delayed fluorescence (TADF), which can significantly increase efficiency of electroluminescent devices and of photocatalytic systems [16,17,18,19].

Perhaps the most popular and readily accessible complexes in this field are heteroleptic mononuclear species [Cu(P^P)(N^N)]^+^, where P^P and N^N are chelate phosphine and diimine ligands, respectively [13]. Bipyridine based N^N ligands control the luminescence properties of [Cu(P^P)(N^N)]^+^ compounds because these ligands compose the frontier orbitals that are directly involved in electronic transitions. On the other hand, the addition of substituents at different positions of the N^N ligand controls the stereochemical and emission behaviour of Cu(I) complex. Indeed, the nature of the N^N ligand, namely, the composition, structure, and electronic properties, determines the photoluminescence characteristics of the [Cu(P^P)(N^N)]^+^ complexes. Decoration of N^N ligands with secondary chemical function provides a synthetic opportunity for the construction of hybrid molecular systems incorporating the Cu(I) chromophore. Therefore, the insight into the influence of the peripheral coordination environment on the luminescence characteristics of Cu(I) complexes is important. In this work we show by the example of a series of Cu(I) complexes with different substituents at the periphery of the N^N ligand that substituents distant from the main action zone can have a significant effect on the physicochemical properties of the system.

## 2. Results and Discussion

### 2.1. Synthesis of Cu(I) Complexes

**Scheme 1:**
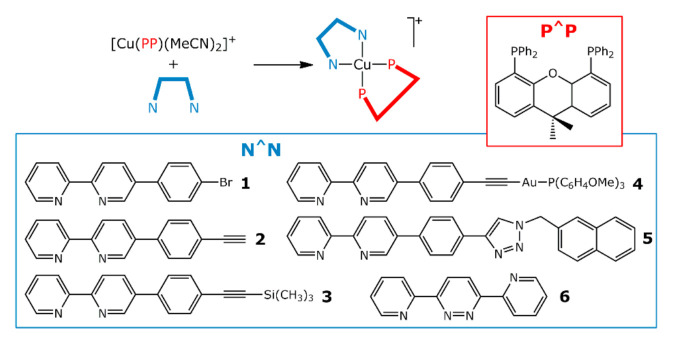
Synthesis of **1**–**6**.

The archetypal Cu(I) heteroleptic cationic complexes [Cu(P^P)(N^N)]^+^ **1**–**6** containing Xantphos and substituted diimine ligand N^N were obtained in the canonical reaction between [Cu(Xantphos)(NCMe)_2_]^+^ and N^N under the mild conditions and in good yields (see Scheme 1 and Experimental Section for details). Xantphos was chosen as P^P ligand due to its steric impact [20,21]. The purity, composition and structures of **1**–**6** were unequivocally established by CHN elemental analysis, 1D and 2D NMR experiments, ESI MS (in positive mode), and single crystal X-ray diffraction analysis (XRD, for complexes **1**, **2** and **6**).

**Figure 1:**
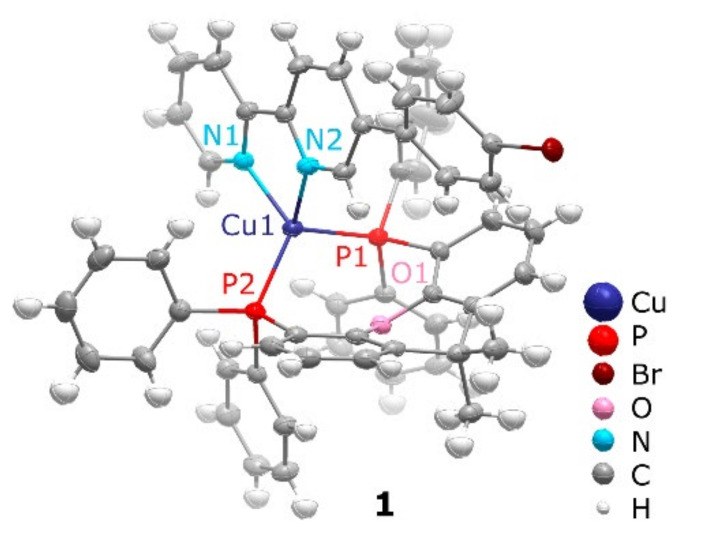
ORTEP view of cation **1**, ellipsoids are shown at 30% probability.

Molecular structures of **1**, **2** and **6** are presented in Figure 1 and Figure S1 (Figure S2 shows DFT-optimized structures of **1**–**6**); crystallographic data are given in Table S1, and selected structural parameters are listed in Table S2. Solid state structures of **1**, **2** and **6** are similar to those of the related heteroleptic [Cu(Xantphos)(N^N)]^+^ complexes bearing substituted 2,2′-bipyridines [22,23,24,25,26,27,28,29,30,31,32,33,34,35,36,37,38,39]. The structures demonstrate the expected chelating mode of both Xantphos and N^N ligands as well as the moderately distorted tetrahedral coordination geometry of the Cu(I) centre (Table S3) [40,41].

Complexes **1**, **2**, on the one hand, and **6,** on the other hand, demonstrate different orientations of the asymmetrical N^N ligand with respect to the Xantphos backbone. The substituted −C_6_H_4_X ring in the 5-position of the bipyridine ligand in **1** and **2** is located over the Xantphos ‘bowl’, while the inverse configuration is found in **6** (Figure S5); however, the coordination geometry of the Cu(I) atom remains almost unchanged (Figure S6). The ability of the [Cu(Xantphos)(N^N)]^+^ cation with an asymmetric N^N ligand to form the pair of different isomers that are involved in dynamic processes such as inversion of the ‘bowl’ linker of Xantphos have been reported in the literature [34,37,38,42]. A preference for a resulting orientation of N^N in the solid state is usually influenced by steric factors, and is determined by repulsion of a substituent with hydrogens of Xantphos phenyl rings [31]. It is interesting that in the case of the 5,6′-Me_2_bpy, the ring with the 5-position substituent is located over the ‘bowl’ linker of Xantphos [30].

The orientation of the N^N ligand in [Cu(Xantphos)(N^N)]^+^ complexes is likely determined by a combination of intra- and intermolecular interactions, with the latter being primarily dependent on the packing of molecules in the crystal. The observed location of aromatic rings of 3,6-di(2-pyridyl)pyridazine (dppn) in relation to the {Cu(Xantphos)} fragment in **6** allows ππ interactions between two cations in the solid state (Figure S7). The distance between parallel planes, which include all C and N atoms of the coordinated {C_4_N_2_} and free {C_5_N} rings of dppn, is 3.530 Å.

**Figure 2:**
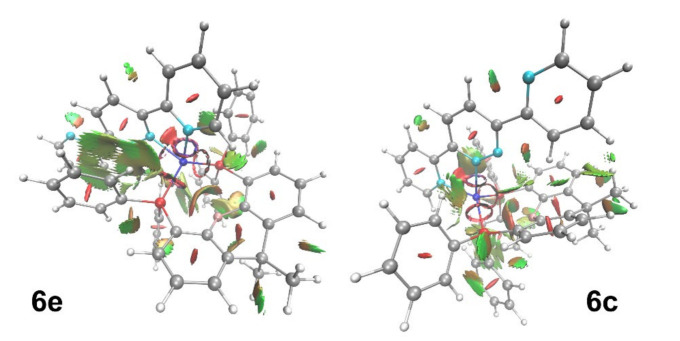
Non-covalent interactions in the optimized structures of **6**. **6e** is experimental orientation determined by XRD; **6c** is abnormal orientation calculated by DFT. Color legend: van der Waals interactions green, steric effect red, hydrogen bonding blue.

The intramolecular interactions, in turn, can be studied in detail using the data obtained from single-molecule DFT calculations. The NCI technique [43] was used to visualize non-covalent interactions in the optimized structures of complex **6**. The anomalous orientation relative to **1, 2** of the N^N ligand in complex **6** observed in experimental studies appears to be the result of strong van der Waals interactions due to the stacking of the rings of the N^N ligand and the phenyl substituent at one of the phosphorous atoms (Figure 2). While this orientation was found to be less favorable in DFT calculations, the energy difference between two possible orientations is very small (ca. 5 kJ/mol); thus, the relative stability of the two isomers can be easily affected by any other external factors.

The composition and structure of **1**–**6** obtained in solution correlates well with the molecular arrangement found for **1**, **2** and **6** in the crystal. The ESI MS spectra of **1**–**6** exhibit the signal of the molecular ion with the composition of [Cu(Xantphos)(N^N)]^+^ accompanied by the signals of a certain expected fragments with isotopic distributions matching those predicted ones (Figures S8 and S9). The formation of homoleptic cations [Cu(N^N)_2_]^+^ and [Cu(Xantphos)_2_]^+^ was not observed [24].

The ^31^P{^1^H} NMR spectra of **1**–**6** display two signals, the singlet and the multiplets assigned to Xantphos and the PF_6_^−^ anion, respectively, with integral intensity relations of 2/1. An additional singlet with a single integral intensity presents in the spectrum of **4** due to the phosphine ligand of the metalloligand Au-epbpy. The resonance of Xantphos phosphorus atoms is located around −12 ppm, which is significantly shifted to the low field region compared to free phosphine, and therefore confirms the coordination of PPh_2_ groups to the Cu(I) centre in solution.

The ^1^H NMR spectroscopic patterns for **1**–**6** are completely compatible with the proposed molecular structures. The complete assignment of proton resonances was performed based on ^1^H^1^H COSY spectra (Figures S10−S17). The number of the signals and their relative integral intensities and multiplicities clearly indicate the presence of the both Xantphos and N^N ligands in the coordination environment of **1**–**6**, as shown in Scheme 1. The ^1^H NMR spectra of **1**–**6** show the set of resonances, which are characteristic for Cu(I)-bound ligands that confirmed the stability of the complexes in solution. Both the ^31^P and ^1^H NMR spectra provide support for the presence of only one conformer in solution.

In contrast to **1**–**5**, complex **6** demonstrates the dynamic flexibility of the ligand environment in solution. Variable-temperature ^1^H NMR spectra were recorded in acetone–d_6_ solution (Figure S18), demonstrating the effect of temperature in both the aromatic and the aliphatic regions. At ambient temperature, most dppn resonances and part resonances of Xantphos are broad while methyl protons of Xantphos collapse, yielding a single signal with 6H intensity. Thus, the coordination environment of **6** undergoes a ‘merry-go-around’ process of ligands together with flipping of the Xantphos ‘bowl’ [34,37,42]. When the temperature decreases, all resonances split, resulting in a set of resolved signals at 233 K corresponding to a single conformer. Such conformational dynamics have been previously described for Xanphos-containing complexes and have been shown to be due to intramolecular conformational dynamics arising from interconversion of enantiomeric forms rather than from dynamic exchange between the two forms [44].

### 2.2. Electrochemical Properties of ***1**−**6***

The electrochemical behaviour of the heteroleptic complexes was investigated by cyclic voltammetry (CV). The voltammetry patterns of compounds **1**−**6** are shown in Figure S19. Two oxidation peaks can be observed on the CVs of all compounds except complex **6,** which correlates with typical voltammograms of [Cu(^t^Bu-Xantphos)(bpy)] complexes [45]. The first peak, observed on CVs of **1**−**6**, is usually attributed to metal-centred processes of the type Cu^+^/Cu^2+^, while the second oxidation processes, visible only for **1**−**5**, are usually ligand centred. The reduction processes for compounds **1**−**5** were poorly resolved, while **6** demonstrates reversible reduction at a potential of −1.7 V. Due to the irreversibility of the oxidation processes, differential pulse voltammetry (DPV) (Figure S19) was used to determine the precise values of the oxidation potentials, and further DPV data will be analyzed. The first oxidation potentials, $E_{1/2}^{\mathrm{ox}}$**(1)** (Table 1), for compounds **1**−**4** and **6** are close to 0.8 V vs. Fc/Fc^+^, while the voltammogram of the oxidation of compound **5** contains a less pronounced peak at 0.72 V. It indicates that the metal-centred oxidation processes of complexes **1**−**6** are similar to each other and the Cu^+^/Cu^2+^ transition potential is close to that observed in similar complexes (0.8 V vs. Fc/Fc^+^) [23,24,25,26,27,29,30,35,37,45,46,47]. The low intensity of the first oxidation peak in the complex **5** indicates that metal oxidation is suppressed, possibly, due to sterical reasons. The second oxidation peak of complexes **1**, **2** and **4, 5** is located in the 1.2−1.4 V range, and corresponds to bpy-type ligand oxidation. No characteristic ligand oxidation peak was observed for compound **6,** which has dppn instead of a bpy-type ligand. The second oxidation process of compound **3** differs from the oxidation of complexes **1, 2** and **4, 5**; on DPV of oxidation of **3**, two ligand oxidation peaks are following the metal-centred process. Thus, the results of the electrochemical experiments clearly indicate the dependence of the physicochemical properties of the complexes on the composition of the diimine ligand, as the oxidation process proceeds with different energy depending on the nature of the substituent at the periphery. The difference in electrochemical behaviour is reflected in the physicochemical properties of the complexes, in particular, their photophysical behaviour, *vide infra*.

### 2.3. Photophysical Properties of ***1**−**6***

**Figure 3:**
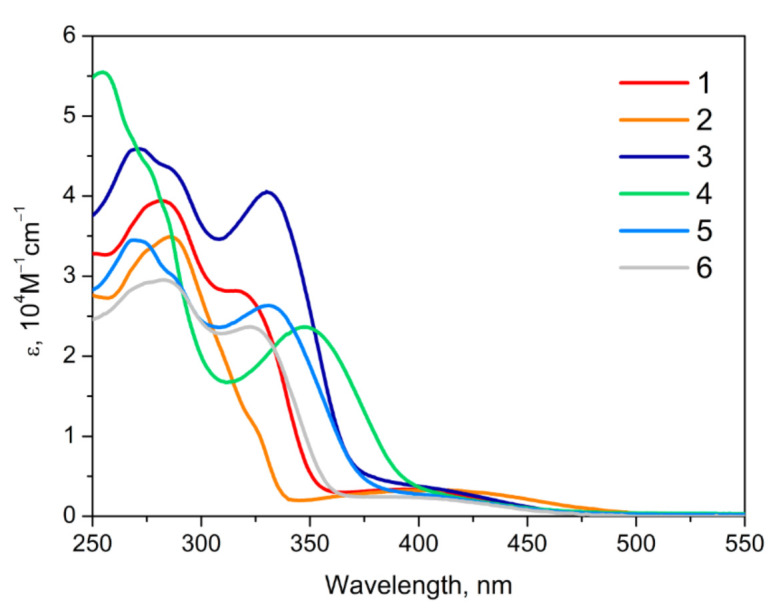
Absorption spectra of **1**−**6** in dichloromethane solution at r.t.

The UV–vis absorption spectra of **1**–**6** (Figure 3, Table 2) are typical for heteroleptic Cu(I) complexes with diimine and phosphine ligands in a coordination sphere, and exhibit strong bands in the range from 250 to 350 nm due to spin-allowed ππ* intraligand (^1^IL) transitions located at the aromatic system of both the N^N and P^P ligands [23,24,25,26,29,30,31,32,33,34,35,37,38,39,48,49]. The intense bands in the 320–350 nm region observed for **1**–**6** can be tentatively assigned to the transitions localized within the {diimine + C_6_H_4_X} motif coordinated to the Cu(I) atom. The broad low-energy band at ca. 400 nm includes the contribution of the metal-to-ligand charge transfer (MLCT) from the metal dπ-orbitals to the π^*^-orbitals of ligands, as suggested by the earlier assignments made for similar complexes [27].

It is worth noting that the nature of the substituents of the N^N ligand influences the absorption profiles of **1**–**6**. The red edge of the IL band assigned to transitions in the N^N ligand undergoes a bathochromic shift, the highest of which corresponds to heterometallic complex **4**. This phenomenon appears due to electronic communication between the chromophoric center and the heavy metal atom through the C≡C bond, and is typical of alkynyl-phosphine Au(I) complexes [50,51]. The extinction coefficients are of the order of 10^4^ M^−1^cm^−1^ and 10^3^ M^−1^cm^−1^ for the high energy and low energy absorption bands, respectively, which is in accordance with the data reported for other complexes [Cu(Xantphos)(N^N)]^+^ [23,24,25,26,29,30,31,32,33,34,35,37,38,39,48]. The empirical assignment of the bands in absorption spectra of 1–6 was confirmed by TDDFT calculations, *vide infra*.

Compounds **1**–**5** demonstrate photoemission in the solid state in the visible region of the spectrum, with the excited state lifetime in the microsecond domain (Table 2). Complex **6** exhibits no emission in either solution or solid state, in contrast to the other transition metals and lanthanide complexes equipped with a dppn ligand [52,53,54].

**Figure 4:**
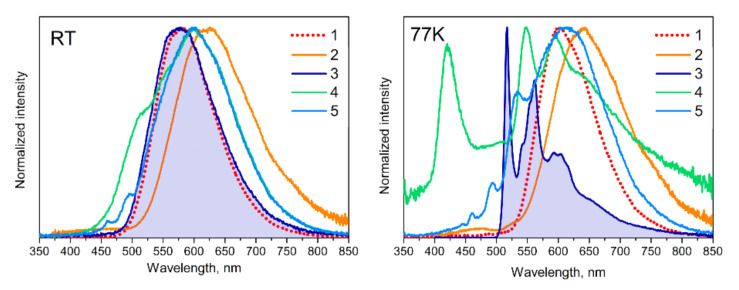
Normalized emission spectra of **1**−**5** in solid state at room temperature and 77 K, λ_exct_ = 351 nm.

The normalized photoemission spectra of **1**–**5** in the solid state are shown in Figure 4, Figures S20 and S21. All compounds are yellow to orange emitters with maxima of luminescence in the range from 580 nm to 620 nm (Table 2 and Table S4), which is similar to the [Cu(Xantphos)(N^N)]^+^ complexes reported to date [23,24,25,26,29,30,31,32,33,34,35,37,38,39,48]. Complexes **1**–**3** show a similar profile of the emission spectra at room temperature, which consists of one moderately wide band. Hybrid systems **4** and **5** are the combination of two emission centers, namely, {Cu(Xantphos)(N^N)} and {C≡C−Au(PR_3_)} (**4**) or the naphthyl fragment, (**5**). As the result, the luminescence spectra of **4** and **5** are superposition of the emission spectra of these two centers, where the red part corresponds to the contribution of the {Cu(Xantphos)(N^N)} fragment. The blue edge of the emission bands shows the vibrational splitting typical of aromatic organic fragments.

The profiles of the emission spectra of compounds **1**–**5** demonstrate different dependence on temperature (Figure 4, Figures S20 and S21). The luminescence energy of **1**, **2** and the red emission band of **5** undergo a bathochromic shift with temperature decrease (Table 2). A silicon atom attached to the conjugated system of the N^N ligand drastically changes the temperature evolution of the emission spectrum of **3**. Emission energy undergoes a hypsochromic shift, and the emission profile of **3** changes its form completely and shows conversion from the structureless band to the band with clear vibronic splitting. This vibronic structuration means the switching of the emissive excited state from ^3^MLCT to ^3^IL, and is a rarely-observed phenomenon in Cu(I) complexes [55].

The temperature behaviour of the emission spectrum of **4** is more sophisticated. The temperature decrease results in (a) the appearance of a new band at 420 nm, (b) the attenuation of the band at 510 nm, and (c) magnification of the structured band at 547 nm. The broad band at ca. 600 nm remains virtually unaffected. The latter component is a result of transitions in the {Cu(Xantphos)(N^N)} fragment, while other components are the result of different transitions realized in the Au(I) moiety, including transitions due to possible intramolecular Au∙∙∙Au interactions in the solid state [56,57].

The broad emission bands of **1**–**3** and the low energy band of **4**, **5** display a significant Stokes shift, and the emission lifetimes are in the microsecond domain (Table 2), which is typical for phosphorescence. However, the bathochromic shift of the emission (except **3**) and a significant elongation of the excited state lifetime with temperature decrease, possibly indicate a TADF nature of the luminescence [17,58]. This observation is in line with recent demonstration of TADF ability in a number [Cu(Xantphos)(N^N)]^+^ complexes [24,25,26,35,38]. The most comprehensive method of arguing for TADF existence is the temperature-dependence of lifetime values in the range from 77 K to room temperature. The lifetime values at different temperatures have to fit the known empirical curve described everywhere [12]. In the case of complexes **1**–**5**, the decay displays a non-single exponential nature (Tables S5 and S6). This phenomenon can be explained, for example, by a disorder of the local environment of the chromophore center due to multiple crystal defects and/or variations in the disposition of the counterions around the cationic coordination centres, which may result in the presence of different deactivation pathways of excited states. This problem prevents an accurate determination of the lifetime, and consequently correct mathematical processing of the obtained experimental results; however for **5** the *τ*_av_ (T) dependence plot provides the TADF character of room temperature emission of **5** (Figure S23). Fitting the dataset of **5** with the corresponding equation results in the following data, obtaining ∆*E*(S_1_−T_1_) = 529 cm^−1^, a fluorescence decay time of *τ*(S_1_) = 16 ns, and a phosphorescence decay time of *τ*(T_1_) = 154.6 µs.

The excitation spectra of **1, 2** and **4, 5** undergo insignificant change at low temperature, indicating that the nature of the emissive excited states remain unchanged in the 77–298 K range (Figures S20 and S21). Significant change in the emission spectra of **3** with temperature decrease reflects a transformation of the excitation spectra that manifests as different excited states operating at low temperature and under ambient conditions. The lifetime emission follows this tendency, and at 77 K is orders of magnitude longer than that obtained at 298 K (τ_av_ 1096.4 μs vs. 2.61 μs), supporting the conclusion regarding different excited states operating at low temperature and at ambient conditions for **3**.

### 2.4. DFT Calculations

**Figure 5:**
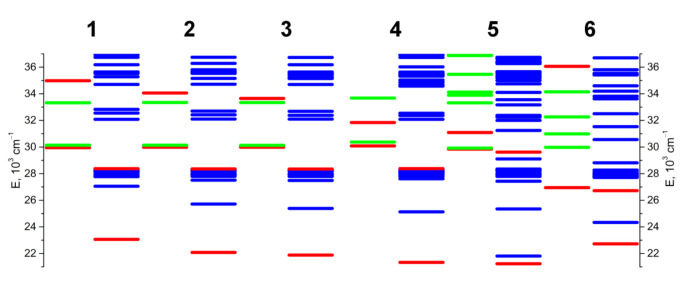
Energy level diagram of the excited states in **1**–**6** obtained from TDDFT calculations. Colour legend shows singlets (green), triplets (blue), and low-lying active states (red).

TDDFT investigations were carried out while taking into account solvent effects without consideration of the solid phase. The obtained photophysical properties of **1**–**6** are in line with the experimental data. TDDFT calculations demonstrate a remarkably similar structure of excited states for complexes **1**–**3**, which is not surprising given the structural similarity of these species (Figure 5 and Figures S24–S29). More interestingly, the excited states for complex **4**, which contain Au(I)-based metalloligand, largely follow the same pattern. Finally, the excited states for complex **5** are similar to **1**–**4**. The lowest singlet excited states for **1**–**5** have essentially the same energy, while the energies of the lowest active singlet state and the lowest triplet states demonstrate a noticeable decrease in this series. An interesting feature of **5** is the existence of two low-lying triplets, while for **1**–**4** the lowest triplet is separated from the higher-lying states by a significant energy gap.

The interpretation of TDDFT absorption spectra is presented at Table S8. It can be concluded that S_4_, which takes part in the excitation process, is the state of interest for complexes **1**−**3**, while S_3_ is responsible for light absorption in **4**−**5** and S_6_ is for **6**.

The properties of the excited states in **1**–**4** suggest a high degree of similarity in the electronic structure of these four complexes. The lowest singlet (S_1_) states reveal MLCT charge transfer from Cu(I) to the N^N ligand, while the triplets (T_1_) are mainly N^N intraligand states. The S_2_ singlet state, which is responsible for weak low-energy absorption, with calculated wavelengths of 329–332 nm and experimental wavelengths of ca. 400 nm, is essentially the same for **1**–**4**, being predominantly of an N^N intraligand nature. The intense absorption band corresponds to an MLCT transition. Thus, the empirical assignment of absorption and emission spectra for **1**–**4** is in agreement with the results of TDDFT calculations. The likely mechanism of luminescence involves the relaxation of the initial excitation down to the S_1_ state, followed by intersystem crossing to one of triplet states with appropriate energy and nature (such states were found to be available for **1**–**4**) and emission from the lowest triplet state.

It should be noted that the −Si(CH_3_)_3_ and −AuP(C_6_H_4_OCH_3_)_3_ substituents in **3** and **4**, respectively, are not involved in low-energy electronic transitions, making the photophysical behavior of the above complexes potentially identical to that of complexes **1** and **2**. While this is mostly true for room-temperature emission, at low temperature the observed luminescence of **3** and **4** is markedly different from other complexes. In spite of the experimentally observed differences in the temperature-dependent photophysical properties of **2** and **3**, from a computational perspective the electronic structure and the nature of excited states of these complexes are nonetheless very similar. Therefore, it can be speculated that the introduction of the −Si(CH_3_)_3_ substituent affects the photophysical properties of the complex via steric and packing effects, rather than via direct influence on the electronic structure of **3**. Despite the formal absence of organogold-based low-lying states in **4**, this complex does have a set of triplet states (T_7_–T_9_, 355–356 nm), which are localized on the {AuP(C_6_H_4_OCH_3_)_3_} moiety and can be populated via deactivation of higher-lying Au(I)-based excited states. In effect, the electronic states associated with the {AuP(C_6_H_4_OCH_3_)_3_} fragment form a quasi-independent subset of states and the fragment itself can be regarded as a quasi-independent emission center, with the above-mentioned triplets being the potentially luminescent states.

In contrast to **3** and **4**, which do not display noticeable differences from **1** and **2** in room-temperature photophysical properties, for **5** the introduction of the ‘click-naphtalene’ substituent into the N^N ligand leads to significant changes in the electronic structure of the latter fragment. The triazole ring appears to be smoothly incorporated into the phenylbipyridine moiety, while the naphtalene fragment seems to be separated from the rest of the N^N ligand in terms of electronic structure. As a result, for complex **5** the lowest intraligand triplet is split into two intraligand triplet states, one of which (T_1_, 469 nm) is localized on naphthalene and the second of which (T_2_, 458 nm) is localized on the triazol-phenylbipyridine fragment. Thus, the N^N ligand in **5** contains two independent chromophoric centres. The singlet states demonstrate photophysical behavior similar to **1**−**4**. It is worth noting that the contribution of the naphthalene fragment can only be found in the lowest triplet (T_1_), and the absence of such a contribution in other low-lying excited states results in the effective isolation of T_1_, making its direct population and consequent naphthalene-based luminescence somewhat problematic. The experimentally observed emission of **5** can be interpreted as a superposition of N^N-based and naphthalene-based luminescence. While such an effect is relatively rare, it is not unique, as the phosphorescence of naphthalene has been observed previously in solid-state photophysical studies [59,60,61,62].

Complex **6** stands out as a distinct exclusion from the series, as the structure of its excited states is markedly different from **1**–**5**. The lowest MLCT/LL’/IL singlet state is similar to the MLCT/LL’ S_1_ states of **1**–**5**; however, it has a noticeably greater wavelength of 371 nm, compared to 332–334 nm for **1**–**5**. This computational result agrees with experimental observations. While the singlet state responsible for absorption is similar to the N^N IL states of other complexes, it has the highest energy of all complexes considered in this study (277 nm vs. 286–322 for other complexes). However, the largest differences are observed for the triplet states. Unlike the T_1_ states of **1**–**5**, which are pure N^N IL, the three lowest triplet states in **6** have a significant MLCT contribution and a minor LL’ contribution. The presence of the ligand-to-ligand contribution may be due to the proximity of the N^N and P^P ligands, which is caused by the unusual orientation of the N^N ligand in **6**. As a result, the excited state deactivation mechanism for complex **6** is likely to be different from that for **1**–**5**, suggesting differences in their observed photophysical behavior. As discussed above, the apparent deactivation mechanism for **1**–**5** assumes relaxation to the lowest excited singlet state, followed by intersystem crossing to the dense group of triplets overlying the lowest triplet state and finally emissive deactivation of the latter state. In contrast, complex **6** has an MLCT/LL’ S_1_ state with a relatively low energy and only three lower-lying triplets, all of which are IL/MLCT. Such a configuration of excited states is far less suitable for efficient intersystem crossing than those observed for **1**–**5**. In combination with the exotic nature of the lowest triplet, this finding can be regarded as an explanation of the observed lack of luminescence for complex **6**.

## 3. Materials and Methods

[Cu(MeCN)_2_Xantphos]PF_6_ [63], 5-(4-Bromophenyl)-2,2′-bipyridine (bpbpy) [64], 5-(4-ethynylphenyl)-2,2′-bipyridine (epbpy) [50], 5-(4-tetramethylsilylethynylphenyl)-2,2′-bipyridine (TMS-epbpy) [50], and 3,6-di(2-pyridyl)pyridazine (dppn) [54] were synthetized according to the published procedures. All other reagents and solvents were purchased from Merck (St. Louis, MO, USA), Alfa Aesar (Ward Hill, Massachusetts, USA), and Fluka (Darmstadt, Germany) and used without further purification. Chemical structures of N^N compounds with abbreviations are shown in Figure S30. The solution ^1^H, ^31^P{^1^H} and ^1^H^1^H COSY NMR spectra were recorded on a Bruker Avance III 400 MHz spectrometer (Billerica, MA, USA). Mass spectra were recorded on a MaXis Bruker Daltonik GmbH instrument (Billerica, MA, USA) in the ESI^+^ mode. Microanalyses were carried out in the analytical laboratory of the University of Eastern Finland using a vario MICRO cube CHNS-analyzer (Elementar, Germany).

**5-(4-(1-(naphtalen-2-ylmethyl)-1H-1,2,3-triazol-4-yl)phenyl)-2,2’-bipyridine, click-naphtalene.** 2-(azidomethyl)naphthalene (100 mg, 0.546 mmol) and epbpy (117 mg, 0.455 mmol) were dissolved in 20 mL of acetone. Sodium ascorbate (360 mg, 1.92 mmol) and CuSO_4_·5H_2_O (227 mg, 0.910 mmol) were dissolved in 5 mL of water and added to the acetone solution. The reaction mixture was heated for 24 h at 70 °C. The solvents were removed in vacuo, and 25 mL of CHCl_3_, 10 mL of water and 5 mL of 5% EDTA water solution were added. The resulting mixture was vigorously stirred for 30 min, then the organic layer was separated and the solvent was removed in vacuo. The resulting beige powder (110 mg, 55%) was washed with pentane and dried. ^1^H NMR (CDCl_3_, 400 MHz, 298 K): δ 8.96 (m, 1H, bpy), 8.72 (d, J 4.0 Hz, 1H, bpy), 8.50 (d, J 8.3 Hz, 1H, bpy), 8.46 (d, J 7.9 Hz, 1H, bpy), 8.07 (dd, J 8.3, 2.4 Hz, 1H, bpy), 7.96 (d, J 8 Hz, 2H, bpy), 7.86 (m, 1H bpy), 7.93–7.82 (m, 3H, naph), 7.77 (s, 1H, triazol), 7.72 (d, J 8 Hz, 2H, bpy), 7.56 (m, 2H, J 8.3 Hz, naph), 7.45 (m, 1H, naph), 7.86 (m, 1H, bpy).

**Au(I) metalloligand, Au-epbpy.** Au(tht)Cl (41 mg, 0.126 mmol) and epbpy (32 mg, 0.126 mmol) were dissolved in 10 mL of acetone and trietlylamine (12 mg, 0.126 mmol) was added to the solution. The resulting suspension was stirred for 30 min and centrifuged. The precipitate was washed with water (2 × 2 mL), acetone (2 × 2 mL) and diethyl ether (2 × 2 mL) and suspended in 5 mL of CH_2_Cl_2_. Tris-(4-metoxyphenyl)phosphine (44 mg, 0.126 mmol) was dissolved in 5 mL of CH_2_Cl_2_ and added to the former suspension. The resulting solution was stirred for 30 min, then passed through alumina and dried in vacuo. Yellow powder (72 mg, 71%). ^1^H NMR (acetone-d_6_, 400 MHz, 298 K): *δ* 9.00 (m, J 2.5 Hz, 1H, bpy), 8.71 (m, J 4.8 Hz, 1H, bpy), 8.57 (d, J 8.4 Hz, 1H, bpy), 8.53 (m, J 8.0 Hz, 1H, bpy), 8.21 (dd, J 8.3, 2.4 Hz, 1H, bpy), 7.95 (td, J 7.7 Hz, 1.8 Hz, 1H, bpy), 7.74 (m, J 8.2 Hz, 2H, bpy), 7.55 (m, 6H, PAr_3_), 7.53 (m, 2H, bpy), 7.43 (m, 1H, bpy), 7.15 (m, 6H, PAr_3_), 3.90 (s, 10H, PAr_3_). ^31^P NMR (acetone-d_6_, 162 MHz, 298 K): *δ* 28.5 (s, 1P, PAr_3_).

**Synthesis of the complexes [Cu(Xantphos)(N^N)]PF_6_, 1**–**6.** All complexes mentioned below were synthetized according to general procedure. [Cu(MeCN)_2_Xantphos]PF_6_ (0.018 mmol) and corresponding N^N ligand (0.018 mmol) were dissolved in dichloromethane (20 mL), the reaction mixture was stirred for 1h at room temperature. Diethyl ether was added to yield crude product as yellow solid. The obtained powder was recrystallized using dichloromethane/diethyl ether mixture, washed with diethyl ether and dried.

**[Cu(Xantphos)(bpbpy)]PF_6_, 1.** Yellow crystals, yield 17.5 mg (88%). ^1^H NMR (acetone-d_6_, 400 MHz, 298 K): δ 9.04 (d, J 4.8 Hz, 1H, bpbpy), 8.77 (d, J 8.4 Hz, 1H, bpbpy), 8.73 (d, J 8.2 Hz, 1H, bpbpy), 8.45 (dd, J 8.4 Hz, J 1.8 Hz, 1H, bpbpy), 8.24 (m, J 7.8 Hz, 1H, bpbpy), 7.94 (m, J 7.9 Hz, 2H, Xantphos), 7.75 (m, 1H, bpbpy), 7.66 (m, 2H, bpbpy), 7.64 (m, 1H, bpbpy), 7.41–7.23 (m, 12H, Xantphos), 7.30 (m, 2H, Xantphos), 7.18 (m, 4H, Xantphos), 6.97 (d, J 8.5 Hz, 2H, bpbpy), 6.84 (m, 4H, Xantphos), 6.61 (m, 2H, Xantphos), 2.00 (s, 3H, Xantphos), 1.64 (s, 3H, Xantphos). ^31^P NMR (acetone-d_6_, 162 MHz, 298 K): δ –11.9 (s, 2P, Xantphos), –144.3 (sp, J 710 Hz, 1P, PF_6_). ESI HRMS (*m/z*): calculated for [C_55_H_43_BrCuN_2_OP_2_]^+^, 953.1322; found 953.1399. Combustion elemental analysis calculated for C_55_H_43_BrCuF_6_N_2_OP_3_: C, 60.15; H, 3.95; N, 2.55. Found: C, 60.33; H, 4.27; N, 2.59%. Single crystals of **1** were obtained by the slow diffusion of solvents through a gas phase at room temperature (CH_2_Cl_2_/hexane).

**[Cu(Xantphos)(epbpy)]PF_6_, 2.** Yellow crystals, yield 16.8 mg (80%). ^1^H NMR (acetone-d_6_, 400 MHz, 298 K): δ 9.07 (m, J 4.9 Hz, 1H, epbpy), 8.78 (d, J 8.4 Hz, 1H, epbpy), 8.74 (d, J 8.0 Hz, 1H, epbpy), 8.46 (dd, J 8.4 Hz, J 2.0 Hz, 1H, epbpy), 8.24 (m, J 7.8 Hz, 1H, epbpy), 7.95 (dd, J 7.7 Hz, J 1.0 Hz, 2H, Xantphos), 7.75 (m, 1H, epbpy), 7.65 (m, 1H, epbpy), 7.58 (m, J 8.5 Hz, 2H, epbpy), 7.41–7.24 (m, 12H, Xantphos), 7.30 (m, 2H, Xantphos), 7.18 (m, 4H, Xantphos), 7.03 (m, J 8.4 Hz, 2H, epbpy), 6.83 (m, 4H, Xantphos), 6.61 (m, 2H, Xantphos), 3.88 (s, 1H, epbpy), 2.02 (s, 3H, Xantphos), 1.63 (s, 3H, Xantphos). ^31^P NMR (acetone-d_6_, 162 MHz, 298 K): δ –11.9 (s, 2P, Xantphos), –144.2 (sp, J 706 Hz, 1P, PF_6_). ESI HRMS (*m/z*): calculated for [C_57_H_44_CuN_2_OP_2_]^+^: 897.2225; found 897.2271. Combustion elemental analysis calculated for C_57_H_46_CuF_6_N_2_OP_3_: C, 64.40; H, 4.22; N, 2.68. Found: C, 64.95; H, 4.40; N, 2.67%. Single crystals of **2** were obtained by the slow diffusion of solvents through a gas phase at room temperature (acetone/hexane).

**[Cu(Xantphos)(TMS-epbpy)]PF_6_, 3.** Yellow crystals, yield 16.6 mg (83%). ^1^H NMR (acetone-d_6_, 400 MHz, 298 K): δ 9.07 (m, J 4.9 Hz, 1H, TMS-epbpy), 8.77 (d, J 8.7 Hz, 1H, TMS-epbpy), 8.73 (d, J 8.2 Hz, 1H, TMS-epbpy), 8.46 (dd, J 8.6 Hz, J 2.0 Hz, 1H, TMS-epbpy), 8.24 (m, J 7.7 Hz, 1H, TMS-epbpy), 7.95 (dd, J_HH_ = 7.8 Hz, J_HH_ = 1.2 Hz, 2H, Xantphos), 7.75 (m, 1H, TMS-epbpy), 7.65 (m, 1H, TMS-epbpy), 7.54 (m, J 8.4 Hz, 2H, TMS-epbpy), 7.41–7.24 (m, 12H, Xantphos), 7.30 (m, 2H, Xantphos), 7.18 (m, 4H, Xantphos), 7.04 (m, J 8.7 Hz, 2H, TMS-epbpy), 6.83 (m, 4H, Xantphos), 6.61 (m, 2H, Xantphos), 2.03 (s, 3H, Xantphos), 1.63 (s, 3H, Xantphos), 0.29 (s, 9H, TMS-epbpy). ^31^P NMR (acetone-d_6_, 162 MHz, 298 K): δ –11.9 (s, 2P, Xantphos), –144.2 (sp, J 700 Hz, 1P, PF_6_). ESI HRMS (*m/z*): calculated for [C_60_H_54_CuN_2_OP_2_Si]^+^: 969.2620; found 969.2899. Combustion elemental analysis calculated for C_60_H_54_CuF_6_N_2_OP_3_Si: C, 64.53; H, 4.66; N, 2.51. Found: C, 63.84; H, 4.81; N, 2.39%.

**[Cu(Xantphos)(Au-epbpy)]PF_6_, 4.** Yellow crystals, yield 17.2 mg (86%). ^1^H NMR (acetone-d_6_, 400 MHz, 298 K): δ 9.43 (m, J 4.8 Hz, 1H, NN-Au), 8.92–8.87 (m, 2H, NN-Au), 8.52 (dd, J 8.6 Hz, 1.7 Hz, 1H, NN-Au), 8.31 (m, J 8.0 Hz, 1H, NN-Au), 8.11 (m, J 7.8 Hz, 2H, Xantphos), 7.73 (m, 1H, NN-Au), 7.54 (m, 6H, NN-Au), 7.48–7.29 (m, 16H, NN-Au, Xantphos), 7.35 (m, 2H, Xantphos), 7.23 (m, 11H, Xantphos), 6.80 (m, 2H, NN-Au), 6.69 (m, 4H, Xantphos), 6.49 (m, 2H, Xantphos), 3.89 (s, 9H, NN-Au), 2.14 (s, 3H, Xantphos), 1.53 (s, 3H, Xantphos). ^31^P NMR (acetone-d_6_, 162 MHz, 298 K): δ 38.0 (s, 1P, PAr_3_), –11.9 (s, 2P, Xantphos), –144.2 (sp, J 701 Hz, 1P, PF_6_). ESI HRMS (*m/z*): calculated for [C_78_H_64_AuCuN_2_O_4_P_3_]^+^: 1445.3040; found 1445.3558. Combustion elemental analysis calculated for C_78_H_64_AuCuF_6_N_2_O_4_P_4_: C, 58.86; H, 4.05; N, 1.76. Found: C, 58.41; H, 4.12; N, 1.70%.

**[Cu(Xantphos)(click-naphtalene)]PF_6_, 5.** Yellow crystals, yield 13.2 mg (66%). ^1^H NMR (acetone-d_6_, 400 MHz, 298 K): δ 9.42 (m, J 4.7 Hz, 1H, click-naphtalene), 8.94–8.87 (m, 2H, click-naphtalene), 8.74 (s, 1H, click-naphtalene), 8.55 (m, J 8.1 Hz, 1H, click-naphtalene), 8.32 (m, J 7.7 Hz, 1H, click-naphtalene), 8.07–7.98 (m, 9H, click-naphtalene), 7.72 (m, 1H, click-naphtalene), 7.62 (m, 2H, click-naphtalene), 7.47 (m, 1H, click-naphtalene), 7.49–7.27 (m, 16H, Xantphos, click-naphtalene), 7.20 (m, 4H, Xantphos), 6.96 (m, J 8.1 Hz, 2H, Xantphos), 6.69 (m, 4H, Xantphos), 6.48 (m, 2H, Xantphos), 5.96 (s, 2H, click-naphtalene), 1.52 (s, 3H, Xantphos). ^31^P NMR (acetone-d_6_, 162 MHz, 298 K): δ –11.9 (s, 2P, Xantphos), –144.2 (sp, J 699 Hz, 1P, PF_6_). ESI HRMS (*m/z*): calculated for [C_68_H_53_CuN_5_OP_2_]^+^: 1080.3021; found 1080.3042. Combustion elemental analysis calculated for C_68_H_53_CuF_6_N_5_OP_3_: C, 66.58; H, 4.36; N, 5.71. Found: C, 65.72; H, 4.40; N, 5.77%.

**[Cu(Xantphos)(dppn)]PF_6_, 6.** Orange crystals, yield 18.0 mg (90%). ^1^H NMR (acetone-d_6_, 400 MHz, 233 K): δ 9.04 (d, J 9.0 Hz, 1H, dppn), 8.96 (d, J 4.8 Hz, 1H, dppn), 8.89 (d, J 9.0 Hz, 1H, dppn), 8.79 (m, 2H, dppn), 8.36 (m, J 7.9 Hz, 1H, dppn), 8.15 (td, J 1.5 Hz, 7.9 Hz 1H, dppn), 7.95 (m, J 7.7 Hz, 2H, Xantphos), 7.89 (d, J 7.9 Hz, 1H, dppn), 7.79 (m, 1H, dppn), 7.65 (m, 1H, dppn), 7.38 (m, 4H, Xantphos), 7.33–7.20 (m, 8H, Xantphos), 7.30 (m, 2H, Xantphos), 7.19–7.07 (m, 8H, Xantphos), 6.62 (m, 2H, Xantphos), 1.81 (s, 3H, Xantphos), 1.78 (s, 3H, Xantphos). ^31^P NMR (acetone-d_6_, 162 MHz, 298 K): –12.9 (s, 2P, Xantphos), –144.3 (sp, J 708 Hz, 1P, PF_6_). ESI HRMS (*m/z*): calculated for [C_53_H_42_CuN_4_OP_2_]^+^: 875.2130; found 875.2142. Combustion elemental analysis calculated for C_53_H_42_CuF_6_N_4_OP_3_·CH_2_Cl_2_: C, 58.52; H, 4.18; N, 5.06. Found: C, 58.67; H, 4.18; N, 5.12%. Single crystals of **6** were obtained by the slow diffusion of solvents through a gas phase at room temperature (CH_2_Cl_2_/hexane).

**X-ray structure determinations.** The crystal structures of **1, 2** and **6** were determined by the means of single crystal XRD analysis using a Rigaku Oxford Diffraction XtaLAB HyPix-3000 diffractometer (London, UK) for data collection at a temperature of 100K. Diffraction data were processed in the *CrysAlisPro* program, version 1.171.39.35a [65]. The unit–cell and refinement parameters are listed in the Table S1, and selected structural parameters are listed in Table S2. The structures were solved using the dual-space algorithm and refined using the *SHELX* programs [66,67] incorporated in the *OLEX2* program package [68]. The unit cells of **1** and **2** contain disordered solvent molecules, which were treated as a diffuse contribution to the overall scattering without specific atom positions by *SQUEEZE/PLATON* [69]. Supplementary crystallographic data for this paper have been deposited at the Cambridge Crystallographic Data Centre and can be obtained free of charge via www.ccdc.cam.ac.uk/structures/ (access on 21 February 2022). **1**: (*R-*3; *a* = 43.8299(4), *c* = 15.1257(1) Å; *γ* = 120°; *V* = 25164.4(5) Å^3^; *Z* = 18; *R*_1_ = 3.7%; CCDC 1980997). **2**: (*C*2*/c*; *a* = 29.2984(2), *b* = 17.3077(1), *c* = 22.0407(1) Å; *β* = 111.155(1)°; *V* = 10423.35(12) Å^3^; *Z* = 8; *R*_1_ = 3.7%; CCDC 1980999). **6**: (*P*2_1_*/n*; *a* = 13.3249(1), *b* = 17.6631(2), *c* = 21.0984(2) Å; *β* = 94.171(1)°; *V* = 4952.55(8) Å^3^; *Z* = 4; *R*_1_ = 4.1%; CCDC 1980998).

**Electrochemical measurements.** Electrochemical experiments were carried out in a 0.01 M solution of the target complex (0.006 M for compound **2**) in freshly distilled DCE, with 0.1 M [^n^Bu_4_N][BF_4_] as a supporting electrolyte, using an Autolab PGSTAT 30 potentiostat in standard argon-purged three-electrode cells with a palladium wire (0.07 cm^2^) and a platinum wire (1.5 cm^2^) as the working and counter electrodes, respectively. A non-aqueous Ag|AgNO_3_, 0.1M TEABF_4_ (CH_3_CN) electrode (MF-2062, Bioanalytical systems) was used for the reference electrode and the potential values were corrected against an Fc/Fc^+^ couple in situ after each measurement. The CV experiments were conducted at a 0.1 V s^−1^ scan rate. DPV measurements were recorded in the anodic direction with a potential step of 5 mV, modulation time of 25 ms, interval time of 500 ms, and pulse amplitude of 25 mV.

**Photophysical measurements.** The UV/vis absorption spectra were recorded using a Shimadu UV-1800 spectrophotometer (Kyoto, Japan) in a 1 cm quartz cuvette (dichloromethane, 10^−5^ M). The excitation and emission spectra for solid samples at room temperature and at 77 K were measured on a Fluorolog 3 (JY Horiba Inc., Kyoto, Japan) spectrofluorimeter. The integration sphere was used to measure the solid-state emission quantum yield. The powder samples were supported on the quartz glass plates. For lifetime measurements in the temperature range of 78–295 K the samples were placed in a cryostat optCRYO 105. Emission spectra were recorded using an HR2000 spectrometer (Ocean Optics, Duiven, Netherlands). A halogen lamp LS-1-CAL (Ocean Optics, Duiven, Netherlands) and a deuterium lamp DH2000 (Ocean Optics, Duiven, Netherlands) were used to calibrate the absolute response of the system in the 200–1100 nm spectral range. A pulse laser (DTL-399QT Laser-export Co., Ltd., Moscow, Russia) with wavelength 351 nm, pulse energy 50 μJ, pulse width 6 ns, repetition rate 0.01–1 kHz, a monochromator MUM (LOMO, bandwidth of slit 1 nm, St. Petersburg, Russia), photon counting head H10682 (Hamamatsu, Hamamatsu City, Japan), and multiple-event time digitizer P7887 (FAST ComTec GmbH, Oberhaching, Germany) were used for lifetime measurements. Amplitude average lifetime *τ_aver_* = ∑*A_i_τ_i_* was calculated according to a previously published method [70].

**Computational details.** Density functional theory (DFT) calculations were performed using a CAM-B3LYP long-range-corrected hybrid functional [71] with the D3 empirical dispersion term [72]. A combination of Pople’s double- and triple-zeta basis sets was used: 6-311+G* for copper and bromine, 6-31+G* for phosphorous, and 6-31G* for all other atoms. For complexes containing the Au(I)-based metalloligand (**4**) the SDD basis set with MWB60 effective core potential [73] was employed for gold atoms. Solvent effects were taken into account by the implicit-solvent polarizable continuum model implemented in the framework of IEF-PCM formalism [74,75] with chloroform as the solvent. Natural transition orbital (NTO) analysis [76] was carried out to investigate the nature of the excited states. Non-covalent intramolecular interactions were studied using the NCI technique [43] as implemented in MultiWFN software [77]. All DFT calculations were carried out using Gaussian 16 [78]. Full geometry optimizations were carried out for isomers of complexes **1**–**6** with different orientations of the N^N ligand. Excited states of the complexes were studied by using time-dependent DFT (TDDFT) calculations. Vertical transition energies were obtained for optimized ground-state structures.

## 4. Conclusions

Heteroleptic mononuclear complexes [Cu(Xantphos)(N^N)]^+^, where N^N are chelate-substituted 5-phenyl-bipyridine, were synthesized and characterized by spectroscopic methods. Using the C≡C bond on the coordination environment periphery, three hybrid molecular systems with −Si(CH_3_)_3_, −Au(PR_3_) and −C_2_HN_3_(CH_2_)C_10_H_7_ fragments were produced. The latter was obtained in the azide–alkyne cycloaddition reaction, demonstrating the high potential of the click reaction for production of hybrid molecules.

The Cu(I) complexes obtained in the present study demonstrate unexpectedly complicated emission behaviour, which was investigated by spectroscopic and computational methods to understand the pathway of energy transfer. It was found that an emission centre based on Cu(I) complex can exhibit luminescence in phosphorescent or TADF modes, including hybrid molecules. At the same time, an −Si(CH_3_)_3_ fragment connected to periphery C≡C bond provokes the switching of emissive excited state from ^3^MLCT to ^3^IL, a rare phenomenon in Cu(I) complexes. Thus, the results obtained in this study demonstrate an efficient approach to the design of new materials, which allows the construction of Cu(I) complexes with controlled optoelectronic properties as an individual compound for the production of desired hybrid molecules and systems.

## Figures and Tables

**Table 1:** Cyclic voltammetric data for **1**–**6** referenced to internal Fc/Fc^+^ = 0 V; DCE (freshly distilled) solutions with [^n^Bu_4_N][BF_4_] as supporting electrolyte and scan rate of 0.1 V s^−1^.

Complex	$E_{1/2}^{\mathrm{ox}}\left(1\right),\;V$	$E_{1/2}^{\mathrm{ox}}\left(2\right),\;V$
**1**	0.83	shoulder at ca. 1.2
**2**	0.81	1.31
**3**	0.87	1.1; 1.5
**4**	0.86	1.29
**5**	0.72	1.41
**6**	0.80	-

**Table 2:** Photophysical properties of **1**−**5** in solid state, λ_exct_ = 351 nm.

	295 K			77 K	
	λ_em_, nm	Φ, %	τ_av_, µs	λ_em_, nm	τ_av_, µs
**1**	580	2.6	4.2	602	26.5
**2**	622	0.6	1.1	640	20.1
**3**	578	0.2	2.6	516 *	1096.4
**4**	510 ^‡^; 600	0.6	3.8	420; 547 ^‡^; 600	268.5
**5**	445,^§^ 600	0.5	1.1	445,^#^ 613	130.0

* Vibronic spacing ca. 1350−1500 cm^−1^. ^‡^ Vibronic spacing ca. 1450 cm^−1^. ^§^ Vibronic spacing ca. 1460 cm^−1^. ^#^ Vibronic spacing ca. 1500 cm^−1^.

## Data Availability

All data reported herein are accompanying the present article.

## Supplementary Materials

The following supporting information can be downloaded at: https://www.mdpi.com/article/10.3390/molecules27072250/s1, X-ray structure determinations; Table S1: Crystallographic data for **1**, **2** and **6**; Table S2: Selected structural parameters of **1**, **2** and **6**; Table S3: Four-coordinate geometry indexes for **1**, **2** and **6**; Table S4: Commission internationale de l’éclairage (CIE 1931) coordinates for **1**−**5** emission at variable temperature; Table S5: Lifetimes τ_i_ (μs) at different temperatures of **1**−**5** in solid state, λ_exct_ = 351 nm; Table S6: Average lifetime τ_aver_^*^ (μs) at different temperatures of **1**−**5** in solid state, λ_exct_ = 351 nm; Table S7: Selected experimental and calculated bond lengths (Å) in **1**, **2** and **6** for two different isomers; Table S8: Active singlet states, corresponding to the most intense absorption band for **1**−**6**, obtained from TDDFT calculations; Figure S1: ORTEP view of cations **2** and **6**, ellipsoids are shown at 30% probability; Figure S2: DFT-optimized structures of **1**−**6**; Figure S3: Molecular view of the cation **1** surrounded by PF_6_^−^ in crystal packing; Figure S4: Molecular view of the compound **1**. F−Br distance is indicated on the picture; Figure S5: The difference in N^N and **P^P** ligands orientation in coordination environment of cations **1**, **2** and **6**; Figure S6: Structures overlay of **1** (yellow), **2** (dark red), and **6** (light blue). Copper and phosphorous atoms of the compounds are used for the procedure; Figure S7: Molecular view of the two cations **6** in crystal packing; Figure S8: Experimental (grey) ESI^+^ MS spectra of **1**−**3** and simulated isotopic patterns of the most intensive signals; Figure S9: Experimental (grey) ESI^+^ MS spectra of **4**−**6** and simulated isotopic patterns of the most intensive signals; Figure S10: ^1^H (top) and ^1^H^1^H COSY (bottom) spectra in aromatic range of ‘click-naphtalene’; Figure S11: ^1^H (top) and ^1^H^1^H COSY (bottom) spectra in aromatic range of Au(I) metalloligand; Figure S12: ^1^H (top) and ^1^H^1^H COSY (bottom) NMR spectra of **1**, aromatic range; Figure S13: ^1^H (top) and ^1^H^1^H COSY (bottom) NMR spectra of **2**, aromatic range; Figure S14: ^1^H (top) and ^1^H^1^H COSY (bottom) NMR spectra of **3**, aromatic range; Figure S15: ^1^H (top) and ^1^H^1^H COSY (bottom) NMR spectra of **4**, aromatic range; Figure S16: ^1^H (top) and ^1^H^1^H COSY (bottom) NMR spectra of **5**, aromatic range; Figure S17: ^1^H (top) and ^1^H^1^H COSY (bottom) NMR spectra of **6**, aromatic range; Figure S18: Variable temperature ^1^H NMR spectra of **6**, acetone-d_6_ Figure S19: (A) Cyclic voltammograms of **1**−**6** in DCE solution with 0.1 M TBATFB as the supporting electrolyte referenced versus a Fc/Fc^+^ couple with a scan rate of 0.1 V s^−1^; (B) background CV of DCE solution of 0.1 M TBATFB; (C) differential pulse voltammogram of **1**−**6**; Figure S20: Normalized excitation (left) and emission (right, λ_exct_ = 351 nm) spectra of **1–2** in solid state at variable temperature; Figure S21: Normalized excitation (left) and emission (right, λ_exct_ = 351 nm) spectra of **3–5** in solid state at variable temperature; Figure S22: Average lifetime τ_aver_ at different temperatures of **1**−**5** in solid state, λ_exct_ = 351 nm; Figure S23: Temperature dependence and fitting curve of the lifetimes observed (τ_aver_) for **5**, λ_exct_ = 351 nm; Figure S24: Energy level diagram and natural transition orbitals (NTOs) for the most important low-lying excited states in **1** as obtained from TDDFT calculations; Figure S25: Energy level diagram and NTOs for the most important low-lying excited states in **2** as obtained from TDDFT calculations; Figure S26: Energy level diagram and NTOs f for the most important low-lying excited states in **3** as obtained from TDDFT calculations; Figure S27: Energy level diagram and NTOs for the most important low-lying excited states in **4** as obtained from TDDFT calculations; Figure S28: Energy level diagram and NTOs for the most important low-lying excited states in **5** as obtained from TDDFT calculations; Figure S29: Energy level diagram and NTOs for the most important low-lying excited states in **6** as obtained from TDDFT calculations; Figure S30: Abbreviations of N^N ligands correlated with compound **1**−**6** numeration.

## Sample Availability

Samples of compounds **1**–**6** are available from the authors.
