# Supplementary material for: The Tail Wags the Dog: The Far Periphery of the Coordination Environment Manipulates the Photophysical Properties of Heteroleptic Cu(I) Complexes
Source: Molecules. 2022 Mar 30;27(7):2250. doi: 10.3390/molecules27072250 (PMC9000333; doi:10.3390/molecules27072250)
Supplement: Supplementary file 1 [file molecules-27-02250-s001.zip › molecules-1629524-supplementary.pdf]

Supplementary information

The tail wags the dog: the far periphery of the coordination environment manipulates the photophysical properties of heteroleptic Cu(I) complexes

Aleksandra Paderina,[a] Alexey Melnikov,[b] Sofia Slavova,[c] Vladimir Sizov,[a] Vladislav Gurzhiy,[d] Stanislav Petrovskii,[a] Maksim Luginin,[a] Oleg Levin,[a] Igor Koshevoy,[e] and Elena Grachova[a*]

[a] *Institute of Chemistry, St Petersburg University, Universitetskii pr. 26, 198504 St. Petersburg, Russia*

[b] *Centre for Nano- and Biotechnologies, Peter the Great St. Petersburg Polytechnic University, 195251 St. Petersburg, Russia*

[c] *Institute of General and Inorganic Chemistry, Bulgarian Academy of Sciences, Sofia, 1113, Bulgaria*

[d] *Institute of Earth Sciences, St Petersburg University, St. Petersburg, 199034 Russia*

[e] *Department of Chemistry, University of Eastern Finland, 80101 Joensuu, Finland*

E-mail: e.grachova@spbu.ru

Content

X-ray structure determinations 4

Table S1. Crystallographic data for **1**, **2** and **6**. 5

Table S2. Selected structural parameters of **1**, **2** and **6**. 6

Table S3. Four-coordinate geometry indexes for **1**, **2** and **6**. 6

Table S4. Commission internationale de l'éclairage (CIE 1931) coordinates for **1-5** emission at variable temperature. 6

Table S5. Lifetimes τ_i (μs) at different temperatures of **1-5** in solid state, λ_{exct} = 351 nm.......7

Table S6. Average lifetime τ_{aver}^* (μs) at different temperatures of **1-5** in solid state, λ_{exct} = 351 nm. 8

Table S7. Selected experimental and calculated bond lengths (Å) in **1**, **2** and **6** for two different isomers. 9

Table S8. Active singlet states, corresponding to the most intense absorption band for **1−6**, obtained from TDDFT calculations. NTO analysis illustrates the nature of the singlets under consideration. 10

Figure S1. ORTEP view of cations **2** and **6**, ellipsoids are shown at 30% probability. 11

Figure S2. DFT-optimized structures of **1-6**. 12

Figure S3. Molecular view of the cation **1** surrounded by PF_6^- in crystal packing................13

Figure S4. Molecular view of the compound **1**. F−Br distance is indicated on the picture. ...13

Figure S5. The difference in **N^N** and **P^P** ligands orientation in coordination environment of cations **1**, **2** and **6**. 14

Figure S6. Structures overlay of **1** (yellow), **2** (dark red), and **6** (light blue). Copper and phosphorous atoms of the compounds are used for the procedure. 15

Figure S7. Molecular view of the two cations **6** in crystal packing. 16

Figure S8. Experimental (grey) ESI^+ MS spectra of **1-3** and simulated isotopic patterns of the most intensive signals. 17

Figure S9. Experimental (grey) ESI^+ MS spectra of **4-6** and simulated isotopic patterns of the most intensive signals. 18

Figure S10. ^{1}H (top) and ^{1}H^{1}H COSY (bottom) spectra in aromatic range of ‘click-naphtalene’. 19

Figure S11. ^{1}H (top) and ^{1}H^{1}H COSY (bottom) spectra in aromatic range of Au(I) metalloligand. 20

Figure S12. ^{1}H (top) and ^{1}H^{1}H COSY (bottom) NMR spectra of **1**, aromatic range. 21

Figure S13. ^{1}H (top) and ^{1}H^{1}H COSY (bottom) NMR spectra of **2**, aromatic range. 22

Figure S14. ^{1}H (top) and $^{1}H^{1}H$ COSY (bottom) NMR spectra of **3**, aromatic range. 23
Figure S15. ^{1}H (top) and $^{1}H^{1}H$ COSY (bottom) NMR spectra of **4**, aromatic range. 24
Figure S16. ^{1}H (top) and $^{1}H^{1}H$ COSY (bottom) NMR spectra of **5**, aromatic range. 25
Figure S17. ^{1}H (top) and $^{1}H^{1}H$ COSY (bottom) NMR spectra of **6**, aromatic range. 26
Figure S18. Variable temperature ^{1}H NMR spectra of **6**, acetone-d_6 27
Figure S19. (A) Cyclic voltammograms of **1**–**6** in DCE solution with 0.1 M TBATFB as the supporting electrolyte referenced versus a Fc/Fc^{+} couple with a scan rate of 0.1 V s^{-1}; (B) background CV of DCE solution of 0.1 M TBATFB; (C) differential pulse voltammogram of **1**–**6**. 28
Figure S20. Normalized excitation (left) and emission (right, λ_{exct} = 351 nm) spectra of **1-2** in solid state at variable temperature 29
Figure S21. Normalized excitation (left) and emission (right, λ_{exct} = 351 nm) spectra of **3-5** in solid state at variable temperature 30
Figure S22. Average lifetime τ_{aver} at different temperatures of **1**–**5** in solid state, λ_{exct} = 351 nm. 31
Figure S23. Temperature dependence and fitting curve of the lifetimes observed (τ_{aver}) for **5**, λ_{exct} = 351 nm. 32
Figure S24. Energy level diagram and natural transition orbitals (NTOs) for the most important low-lying excited states in **1** as obtained from TDDFT calculations. ...33
Figure S25. Energy level diagram and NTOs for the most important low-lying excited states in **2** as obtained from TDDFT calculations. 34
Figure S26. Energy level diagram and NTOs f for the most important low-lying excited states in **3** as obtained from TDDFT calculations. 35
Figure S27. Energy level diagram and NTOs for the most important low-lying excited states in **4** as obtained from TDDFT calculations. 37
Figure S28. Energy level diagram and NTOs for the most important low-lying excited states in **5** as obtained from TDDFT calculations. 38
Figure S29. Energy level diagram and NTOs for the most important low-lying excited states in **6** as obtained from TDDFT calculations. 39
Figure S30. Abbreviations of N^N ligands correlated with compound **1**–**6** numeration. 40
References 40

X-ray structure determinations

The crystal structures of **1, 2** and **6** were determined by the means of single crystal X-ray diffraction analysis. Crystals were fixed on a micro mounts and the diffraction data have been collected on the Rigaku Oxford Diffraction diffractometer XtaLAB HyPix-3000 diffractometer and measured using monochromated microfocused Cu*Kα* radiation at a temperature of 100K. Data were integrated and corrected for background, Lorentz, and polarization effects. An empirical absorption correction based on spherical harmonics implemented in the SCALE3 ABSPACK algorithm was applied in *CrysAlisPro* program.[1] The unit-cell parameters (Tables S1) were refined by the least-squares techniques. The structures were solved by dual-space algorithm and refined using the *SHELX* programs[2,3] incorporated in the *OLEX2* program package.[4] The final models included coordinates and anisotropic displacement parameters for all non-H atoms. The carbon-bound H atoms were placed in calculated positions and were included in the refinement in the 'riding' model approximation, U_{iso}(H) set to 1.5U_{eq}(C) and C–H 0.96 Å for the CH_3 groups, U_{iso}(H) set to 1.2U_{eq}(C) and C–H 0.97 Å for the CH_2 groups, and U_{iso}(H) set to 1.2U_{eq}(C) and C–H 0.93 Å for the CH groups. The unit cells of **1** and **2** contain disordered solvent molecules which have been treated as a diffuse contribution to the overall scattering without specific atom positions by *SQUEEZE/PLATON*.[5] The total Potential Solvent Accessible Void Vol in **1** is 2903 Å^3 and electron Count Voids / Cell = 480 that is approximately equal to 0.5 hexane molecule per formula unit. The total Potential Solvent Accessible Void Vol in **2** is 1226 Å^3 and electron Count Voids / Cell = 32 that is approximately equal to 0.2 acetone molecule per formula unit. Supplementary crystallographic data for this paper have been deposited at Cambridge Crystallographic Data Centre (CCDC 1980997-1980999) and can be obtained free of charge via www.ccdc.cam.ac.uk/structures/.

Additionally, the cation of **1** in the crystal cell is surrounded by five anions PF_6^- that forms a 'cocoon' around of the complex by multiple F−H and F−Br interactions (Figure S3). The F−H distance lies in the range from 2.359 to 2.663 Å and F−Br distance is 3.200(3) Å (Table S2 and Fig. S4) and indicates non-covalent interactions (hydrogen/halogen bonding), which govern the crystal-packing mode. Potentially, the oxygen atom in the linker of Xantphos ligand is a donor site but Cu−O distances are too long to be considered as meaningful interactions (Table S2) [6]. In contrast to some other reported $[Cu(Xantphos)(N\text{^}N)]^+$ compounds [6–14], a face-to-face π-stacking interactions between phenyl rings of the two PPh_2 units are not observed in **1**, **2** and **6**. The angles between the planes of the phenyl rings are larger than 39°, and the centroid−centroid distances exceed 3 Å.

Table S1. Crystallographic data for **1**, **2** and **6**.

**Compound**	**1**	**2**	**6**
Formula	$C_{55}H_{43}BrCuN_2OP_2$, PF_6, $(CH_2Cl_2)_{0.32}$	$C_{57}H_{44}CuN_2OP_2$, PF_6	$C_{53}H_{42}CuN_4OP_2$, PF_6, CH_2Cl_2
Crystal System	Trigonal	Monoclinic	Monoclinic
a (Å)	43.8299(4)	29.2984(2)	13.3249(1)
b (Å)	43.8299(4)	17.3077(1)	17.6631(2)
c (Å)	15.1257(1)	22.0407(1)	21.0984(2)
α (°)	90	90	90
β (°)	90	111.155(1)	94.171(1)
γ (°)	120	90	90
V (Å^3)	25164.4(5)	10423.35(12)	4952.55(8)
Molecular weight	1125.22	1043.39	1106.28
Space group	R–3	$C2/c$	$P2_1/n$
μ (mm^{-1})	2.976	1.988	3.106
Temperature (K)	100(2)	100(2)	100(2)
Z	18	8	4
D_{calc} (g/cm^3)	1.337	1.330	1.484
Crystal size (mm^3)	0.12 × 0.10 × 0.09	0.09 × 0.06 × 0.04	0.14 × 0.10 × 0.07
Radiation	Cu$K\alpha$	Cu$K\alpha$	Cu$K\alpha$
Total reflections	30550	87250	30503
Unique reflections	10547	9890	9372
Angle range 2θ (°)	4.03–140.00	6.04–140.00	7.61–140.00
Reflections with $\|F_o\| \geq 4\sigma_F$	9656	9271	8423
R_{int}	0.0208	0.0253	0.0420
R_σ	0.0196	0.0121	0.0368
R_1 ($\|F_o\| \geq 4\sigma_F$)	0.0373	0.0370	0.0405
wR2 (|Fo| ≥ 4σF)	0.1029	0.1015	0.1084
R1 (all data)	0.0399	0.0390	0.0448
wR2 (all data)	0.1047	0.1031	0.1118
S	1.056	1.036	1.039
ρ_{min}, ρ_{max}, e/Å^3	–0.448, 1.068	–0.486, 0.583	–1.376, 0.847
CCDC	1980997	1980999	1980998

$R_1 = \Sigma\|\|F_o\| - \|F_c\|\|/\Sigma\|F_o\|$; $wR_2 = \{\Sigma[w(F_o^2 - F_c^2)^2]/\Sigma[w(F_o^2)^2]\}^{1/2}$; $w = 1/[\sigma^2(F_o^2)+(aP)^2 + bP]$, where $P = (F_o^2 + 2F_c^2)/3$; $s = \{\Sigma[w(F_o^2 - F_c^2)]/(n - p)\}^{1/2}$ where n is the number of reflections and p is the number of refinement parameters.

Table S2. Selected structural parameters of **1**, **2** and **6**.

	**1**	**2**	**6**
	**Bond lengths and distances, Å**		
Cu1−N1	2.0457(18)	2.0468(16)	2.0696(18)
Cu1−N2	2.0809(17)	2.0572(15)	2.0458(18)
Cu1−P1	2.2710(6)	2.2268(5)	2.2579(6)
Cu1−P2	2.2347(6)	2.2592(5)	2.2351(6)
Cu1−O1	3.144(1)	3.114(1)	3.138(1)
F3−Br1	3.200(3)	-	-
	**Bond angles, °**		
N1−Cu1−N2	79.81(7)	80.25(6)	79.93(7)
P1−Cu1−P2	115.42(2)	121.03(2)	116.85(2)
C53−F3−Br1	134.5(1)	-	-
P3−F3−Br1	156.4(1)	-	-
	**Dihedral angles, °**		
N1N2−P1P2	89.93(6)	87.64(5)	75.17(5)

Table S3. Four-coordinate geometry indexes for **1**, **2** and **6**.

**Complex**	**α**	**β**	**τ_4**
**1**	115,42	123,66	0,86
**2**	121,02	124,37	0,81
**6**	117,11	128,98	0,81

$$\tau_4 = (360° - (\alpha + \beta))/141°$$ [15,16]

Table S4. Commission internationale de l'éclairage (CIE 1931) coordinates for **1-5** emission at variable temperature.

**Compound**		**78 K**		**295 K**	
		**x**	**y**	**x**	**y**
[Cu(Xantphos)bpbpy]PF_6	**1**	0.5798	0.4068	0.4959	0.4886
[Cu(Xantphos)epbpy]PF_6	**2**	0.5757	0.3776	0.5597	0.4204
[Cu(Xantphos)TMS-epbpy]PF_6	**3**	0.4260	0.5522	0.4929	0.4879
[Cu(Xantphos)Au-epbpy]PF_6	**4**	0.3969	0.3707	0.4560	0.4273
[Cu(Xantphos)click-naphtalene]PF_6	**5**	0.4889	0.4272	0.5023	0.4396

Commission internationale de l'éclairage (CIE 1931) coordinates was calculated from photoluminescence data by using ColorCalculator OSRAM SYLVANIA software, version 7.77.

Table S5. Lifetimes τ_i (μs) at different temperatures of **1-5** in solid state, λ_{exct} = 351 nm.

**T, K**	**1**	**2**	**3**	**4***	**5***
**295**	1.80 (0.26)<br>5.10 (0.74)	0.21 (0.10)<br>0.86 (0.59)<br>1.87 (0.31)	0.67 (0.18)<br>3.03 (0.82)	0.50 (0.33)<br>2.09 (0.48)<br>14.07 (0.19)	0.15 (0.08)<br>0.66 (0.43)<br>1.59 (0.49)
**270**	2.16 (0.25)<br>6.12 (0.75)	–	0.83 (0.18)<br>3.90 (0.82)	0.64 (0.32)<br>2.73 (0.46)<br>17.37 (0.22)	0.18 (0.08)<br>0.84 (0.45)<br>2.07 (0.47)
**265**	–	0.22 (0.06)<br>0.92 (0.45)<br>2.00 (0.49)	–	–	–
**240**	9.60 (0.48)<br>1.34 (0.07)<br>4.84 (0.45)	–	–	1.02 (0.32)<br>4.61 (0.42)<br>32.57 (0.26)	0.30 (0.09)<br>1.55 (0.55)<br>4.04 (0.37)
**230**	–	0.46 (0.06)<br>2.15 (0.50)<br>4.66 (0.44)	6.44 (0.83)<br>1.25 (0.17)	–	–
**200**	1.32 (0.02)<br>7.10 (0.27)<br>14.09 (0.71)	0.85 (0.09)<br>8.00 (0.31)<br>3.71 (0.60)	25.27 (0.18)<br>13.01 (0.82)	1.98 (0.34)<br>11.21 (0.39)<br>82.98 (0.28)	0.54 (0.09)<br>2.93 (0.57)<br>7.93 (0.34)
**170**	2.43 (0.04)<br>9.20 (0.33)<br>18.32 (0.63)	1.69 (0.14)<br>6.39 (0.70)<br>15.06 (0.16)	4.25 (0.17)<br>25.48 (0.83)	3.46 (0.30)<br>22.81 (0.36)<br>166.81 (0.34)	0.81 (0.09)<br>4.45 (0.57)<br>12.91 (0.34)
**140**	2.31 (0.04)<br>8.98 (0.34)<br>19.52 (0.62)	2.37 (0.15)<br>9.00 (0.70)<br>23.43 (0.15)	4.20 (0.17)<br>25.97 (0.83)	3.95 (0.22)<br>29.36 (0.33)<br>230.76 (0.45)	1.28 (0.11)<br>6.84 (0.62)<br>22.4 (0.27)
**110**	2.34 (0.03)<br>11.21 (0.31)<br>22.79 (0.66)	4.02 (0.25)<br>14.06 (0.70)<br>60.14 (0.05)	20.32 (0.15)<br>206.14 (0.85)	4.91 (0.21)<br>36.85 (0.31)<br>283.21 (0.48)	5.51 (0.44)<br>20.42 (0.48)<br>286.68 (0.08)
**90**	2.95 (0.02)<br>17.33 (0.35)<br>30.29 (0.63)	4.73 (0.26)<br>16.10 (0.69)<br>76.41 (0.05)	301.27 (0.48)<br>19.56 (0.06)<br>872.44 (0.46)	5.53 (0.21)<br>44.68 (0.30)<br>326.01 (0.49)	9.95 (0.59)<br>37.52 (0.30)<br>690.63 (0.11)
**78**	2.31 (0.02)<br>31.56 (0.66)<br>17.54 (0.33)	6.43 (0.34)<br>19.88 (0.62)<br>131.4 (0.04)	37.20 (0.06)<br>629.10 (0.55)<br>1920.59 (0.39)	7.28 (0.19)<br>58.01 (0.23)<br>435.26 (0.58)	10.62 (0.57)<br>39.81 (0.30)<br>828.19 0.13)

* For **4** and **5** measurements have been performed at a wavelength corresponding to the emission of the {Cu(Xantphos)(N^N)} fragment.

Table S6. Average lifetime τ_{aver}* (μs) at different temperatures of **1-5** in solid state, λ_{exct} = 351 nm.

**T, K**	**1**	**2**	**3**	**4****	**5****
**295**	4.23	1.12	2.61	3.82	1.07
**270**	5.13	–	3.34	5.32	1.37
**265**	–	1.40	–	–	–
**240**	6.87	–	–	10.81	2.36
**230**	–	3.14	5.55	–	–
**200**	11.92	4.77	11.11	27.99	4.42
**170**	14.75	7.16	21.80	65.78	6.98
**140**	15.23	10.16	22.25	113.80	10.46
**110**	18.69	14.09	177.44	148.83	35.17
**90**	25.21	16.43	546.61	174.99	93.67
**78**	26.51	20.10	1096.4	268.53	129.97

* Amplitude average lifetime $\tau_{aver} = \sum A_i \tau_i$ was calculated according to published method.[17]

* For **4** and **5** measurements have been performed at a wavelength corresponding to the emission of the {Cu(Xantphos)(N^N)} fragment.

Table S7. Selected experimental and calculated bond lengths (Å) in **1**, **2** and **6** for two different isomers.

	**1**			**2**			**6**		
	**experiment**	**calcd normal**	**calcd abnormal**	**experiment**	**calcd normal**	**calcd abnormal**	**experiment**	**calcd normal**	**calcd abnormal**
**Bond lengths and distances, Å**									
Cu1−N1	2.0457(18)	2.070	2.113	2.0468(16)	2.070	2.092	2.0696(18)	2.114	2.114
Cu1−N2	2.0809(17)	2.114	2.084	2.0572(15)	2.113	2.117	2.0458(18)	2.084	2.084
Cu1−P1	2.2710(6)	2.269	2.291	2.2268(5)	2.273	2.313	2.2579(6)	2.291	2.291
Cu1−P2	2.2347(6)	2.273	2.257	2.2592(5)	2.269	2.244	2.2351(6)	2.259	2.259
Cu1−O1	3.144(1)	3.167	3.188	3.114(1)	3.166	3.200	3.138(1)	3.178	3.178
**Bond angles, °**									
N1−Cu1−N2	79.81(7)	78.660	78.498	80.25(6)	78.696	77.983	79.93(7)	78.534	78.534
P1−Cu1−P2	115.42(2)	115.778	115.840	121.03(2)	115.769	116.287	116.85(2)	115.694	115.694
**Dihedral angles, °**									
N1N2−P1P2	89.93(6)	88.955	75.460	87.64(5)	89.335	82.606	75.17(5)	73.423	75.614

Table S8. Active singlet states, corresponding to the most intense absorption band for **1**−**6**, obtained from TDDFT calculations. NTO analysis illustrates the nature of the singlets under consideration.

	**S_n state**	**E, eV**	**E, nm**	***f***	**NTO**	**NTO***
**1**	S_4	4.3	286	0.82	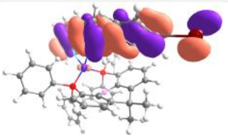	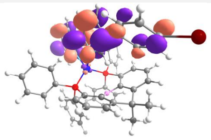
**2**	S_4	4.2	294	1.01	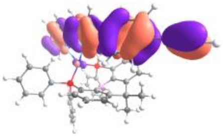	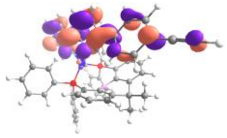
**3**	S_4	4.2	297	1.18	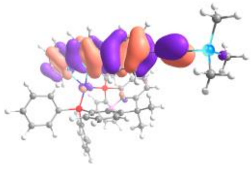	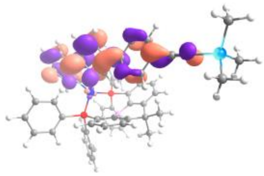
**4**	S_3	3.9	314	1.45	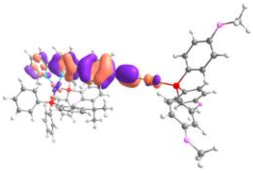	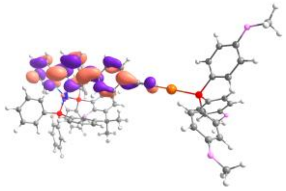
**5**	S_3	4.1	301	1.12	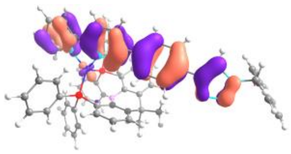	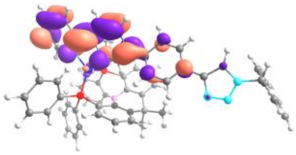
**6**	S_6	4.5	277	0.47	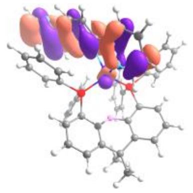	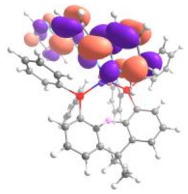

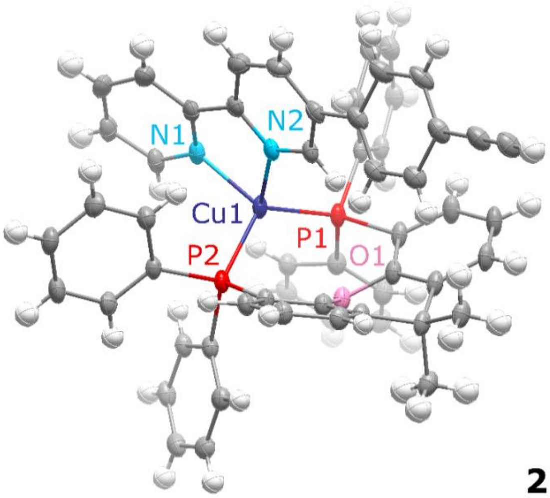

2

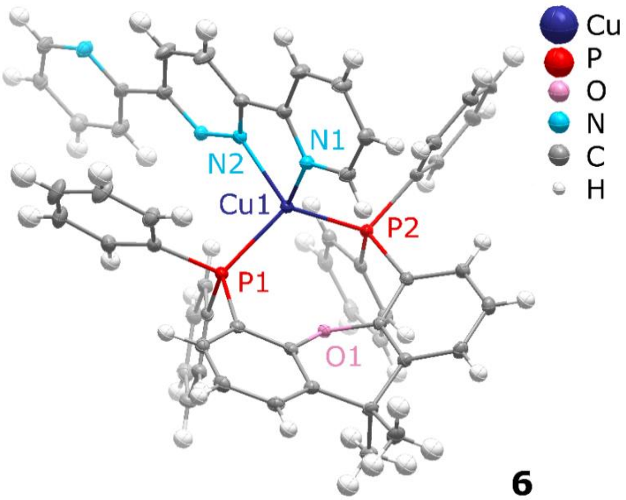

6

Figure S1. ORTEP view of cations **2** and **6**, ellipsoids are shown at 30% probability.

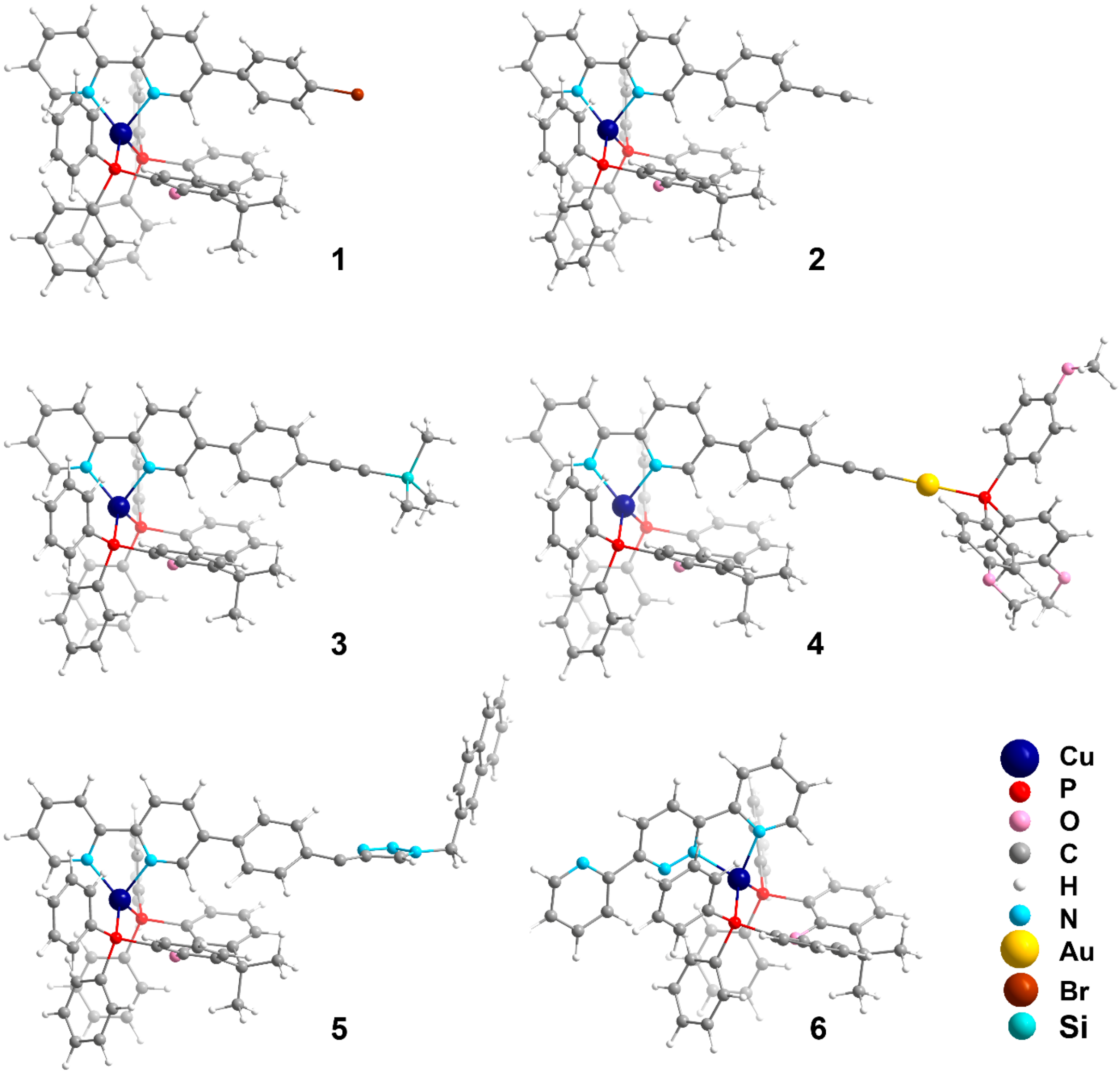

Figure S2. DFT-optimized structures of **1-6**.

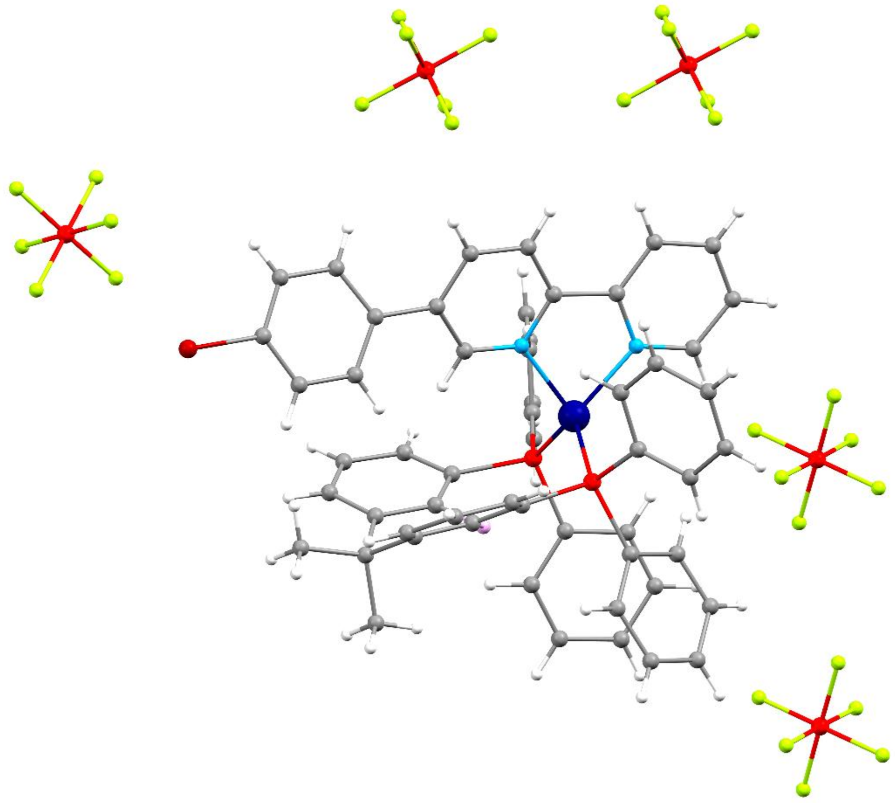

Figure S3. Molecular view of the cation **1** surrounded by PF_6^- in crystal packing.

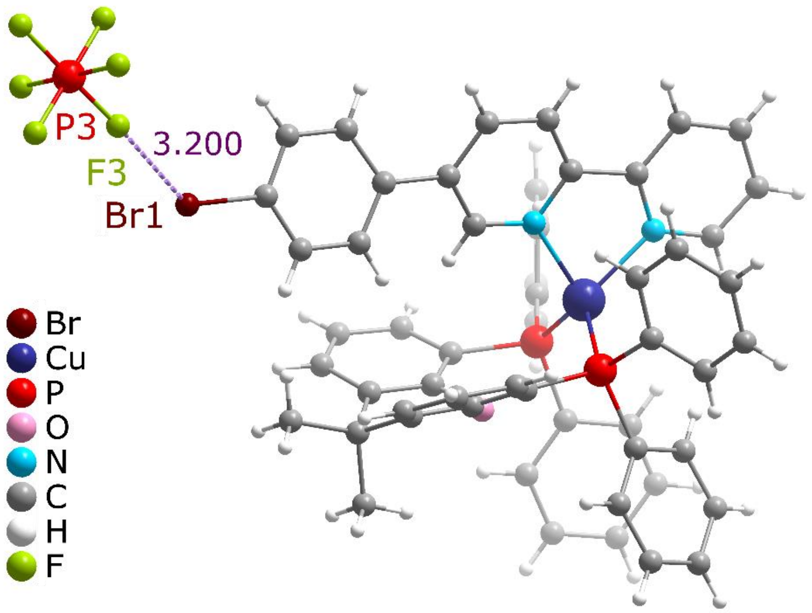

Figure S4. Molecular view of the compound **1**. F−Br distance is indicated on the picture.

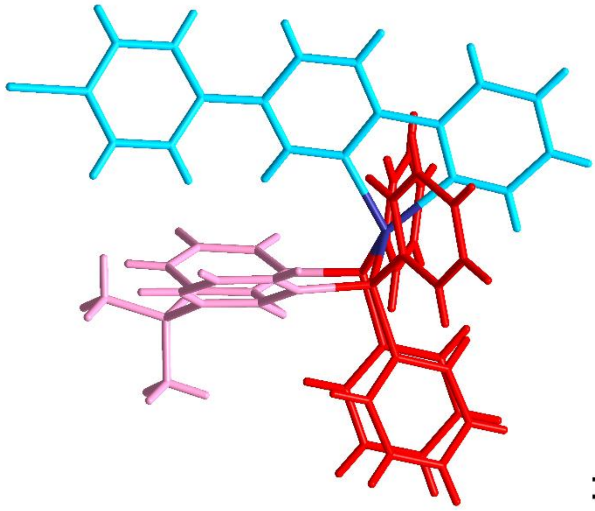

1

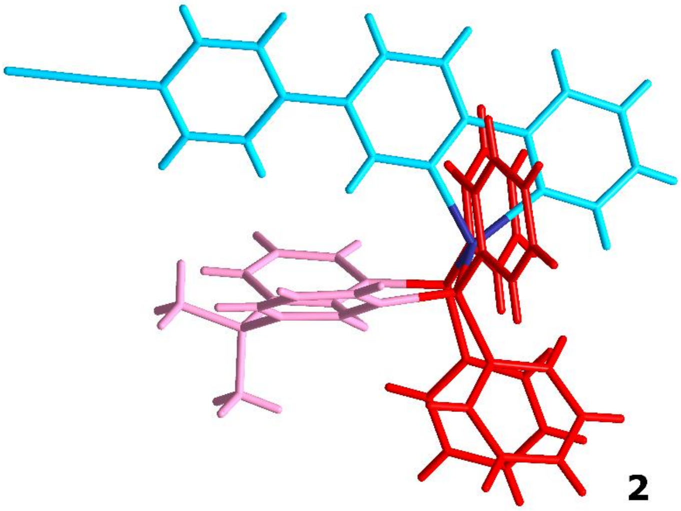

2

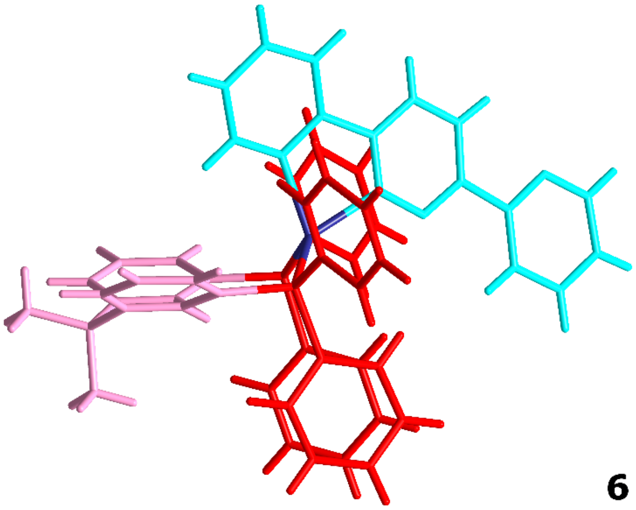

6

Figure S5. The difference in **N^N** and **P^P** ligands orientation in coordination environment of cations **1**, **2** and **6**.

Colour legend: **N^N** sky blue; the linker between P atoms of **P^P** pink; phosphorus atoms and Ph rings of **P^P** red; copper indigo.

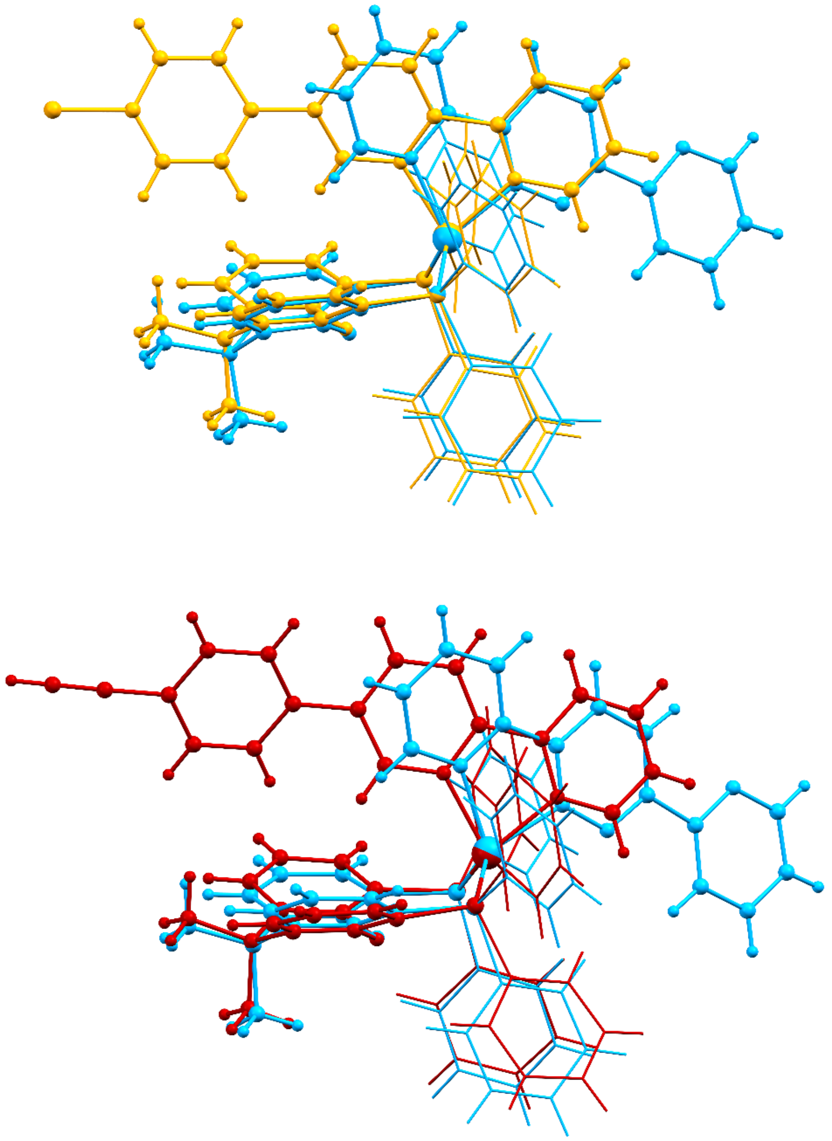

Figure S6. Structures overlay of **1** (yellow), **2** (dark red), and **6** (light blue). Copper and phosphorous atoms of the compounds are used for the procedure.

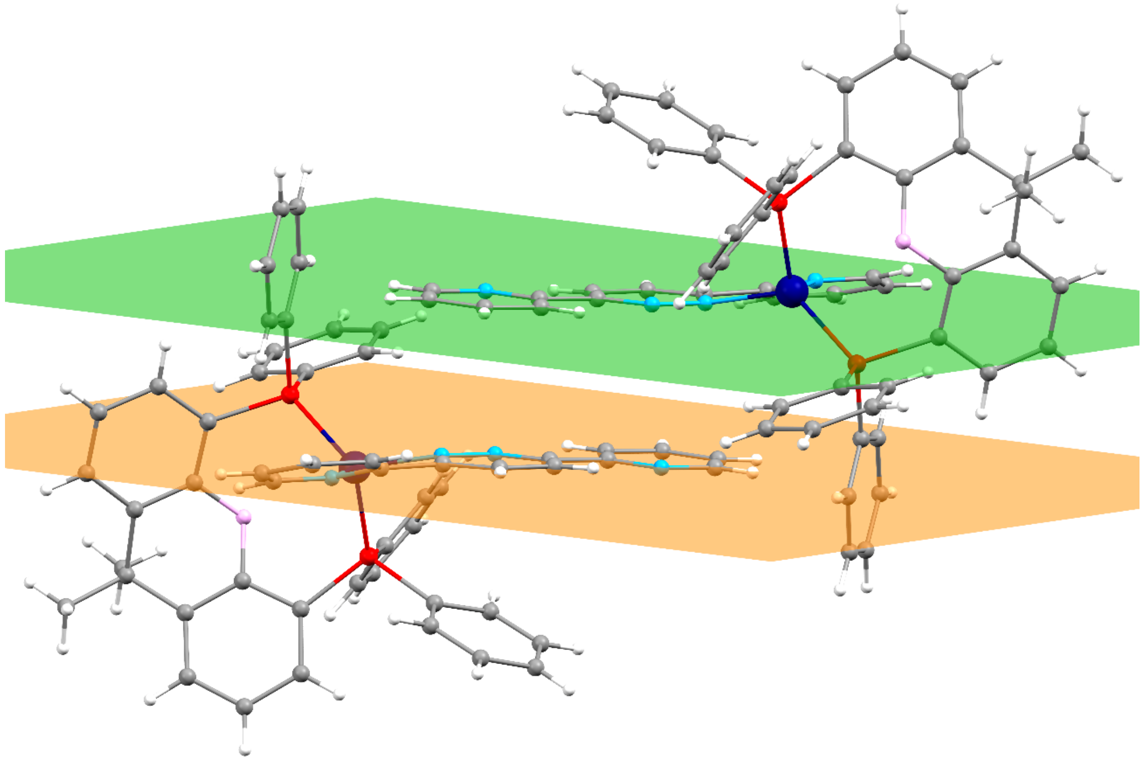

Figure S7. Molecular view of the two cations **6** in crystal packing.

The parallel planes include all C and N atoms of coordinated C_4N_2 and free C_5N rings of ligand **dppn**. The distance between planes is 3.530 Å.

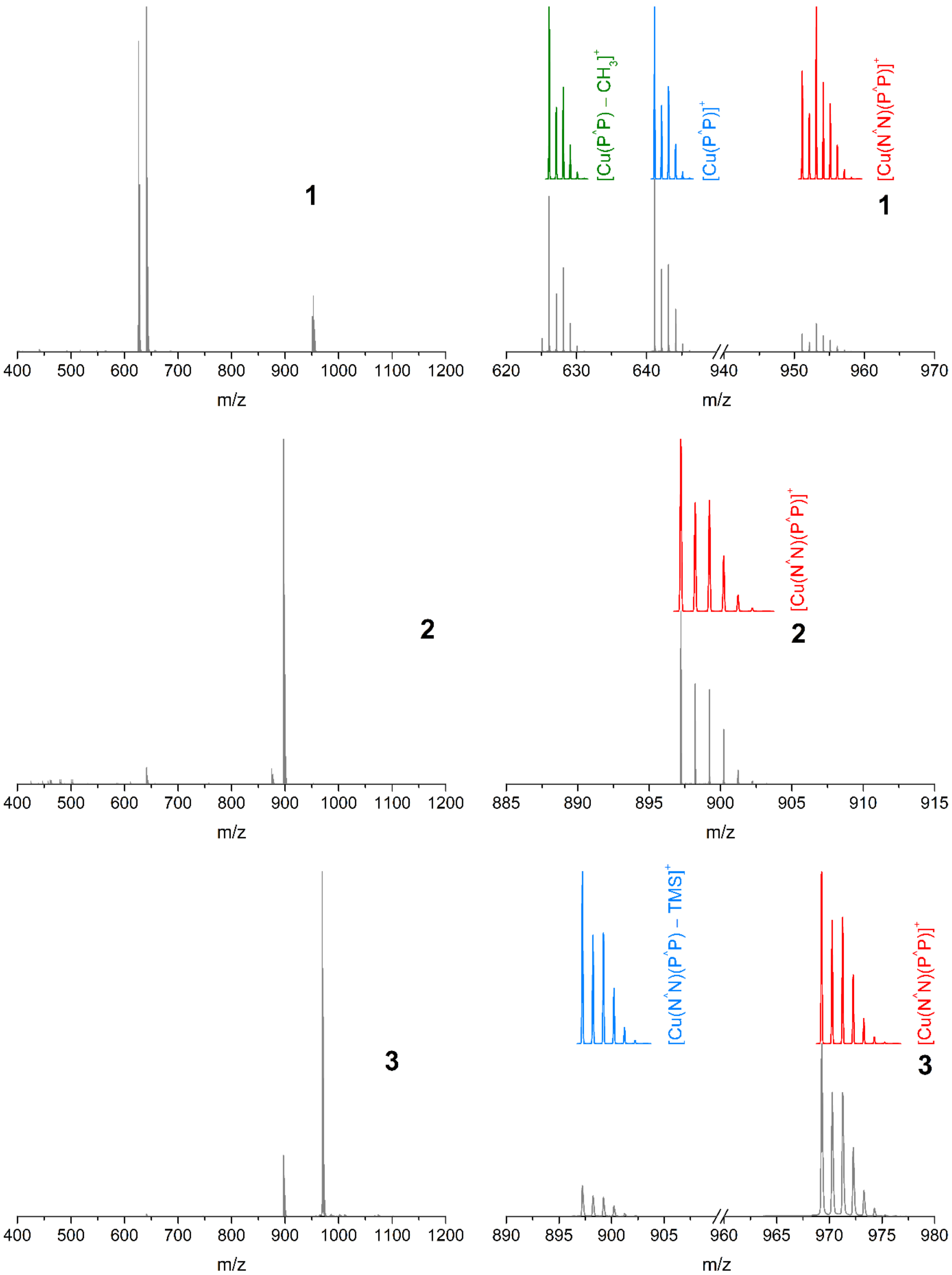

Figure S8. Experimental (grey) ESI^+ MS spectra of **1-3** and simulated isotopic patterns of the most intensive signals.

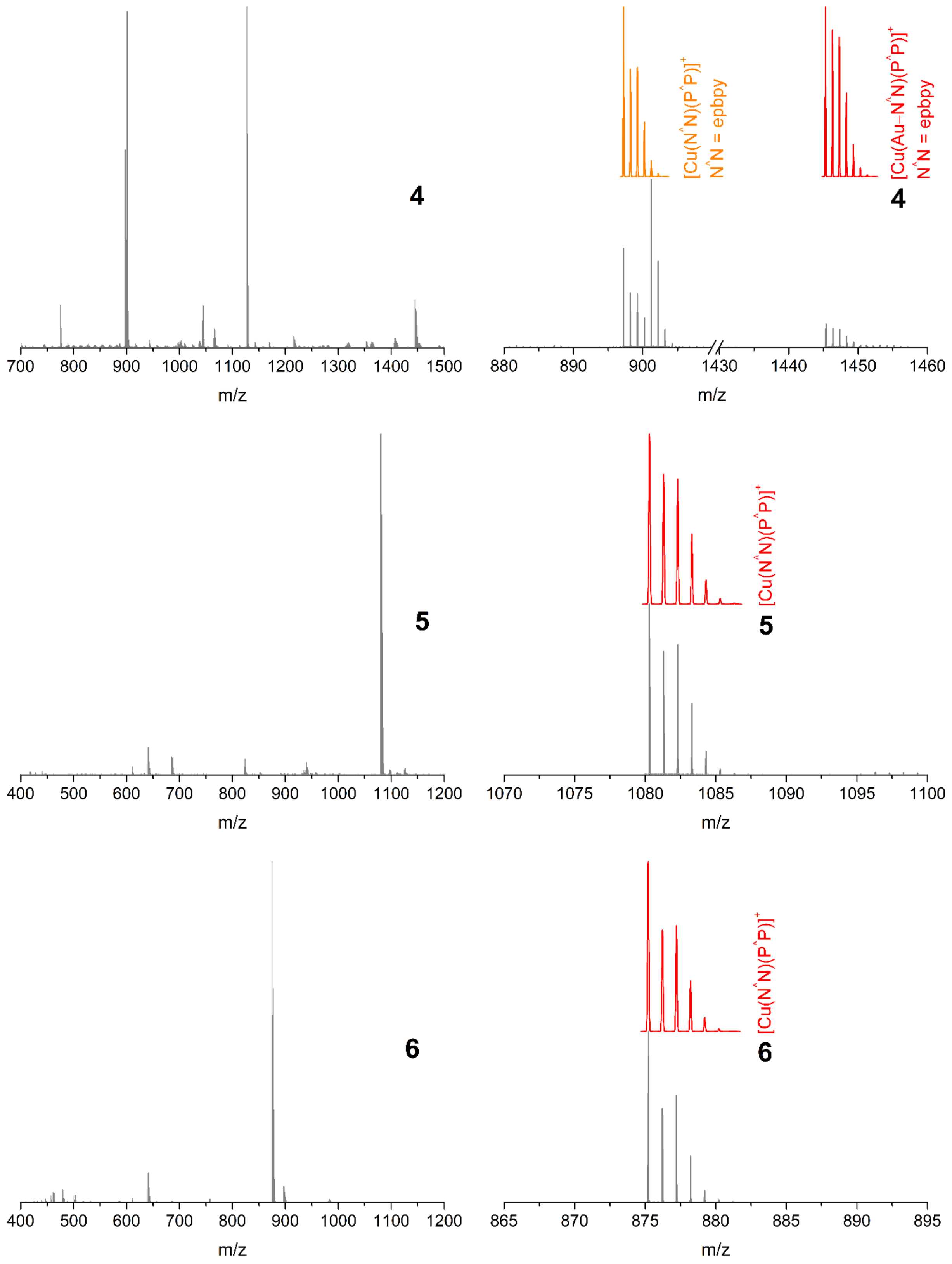

Figure S9. Experimental (grey) ESI^+ MS spectra of **4-6** and simulated isotopic patterns of the most intensive signals.

'click-naphtalene', chloroform-d3 ●, 298 K

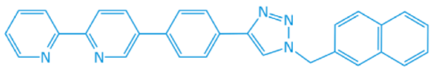

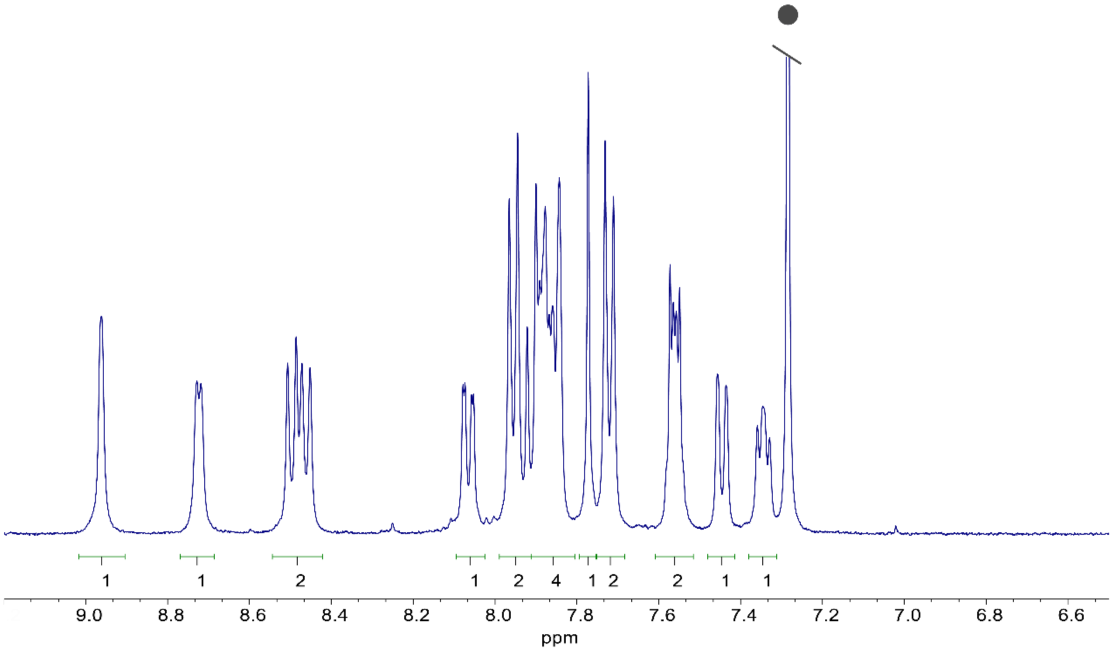

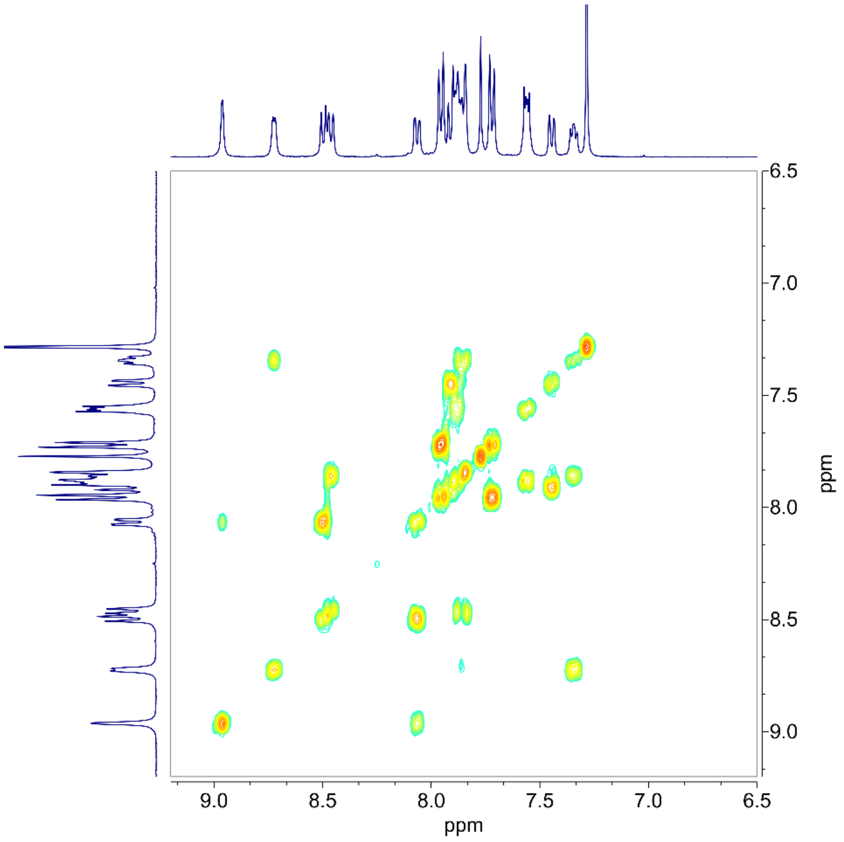

Figure S10. ^{1}H (top) and $^{1}H^{1}H$ COSY (bottom) spectra in aromatic range of 'click-naphtalene'.

Au(I) metalloligand, acetone-d_6, 298 K

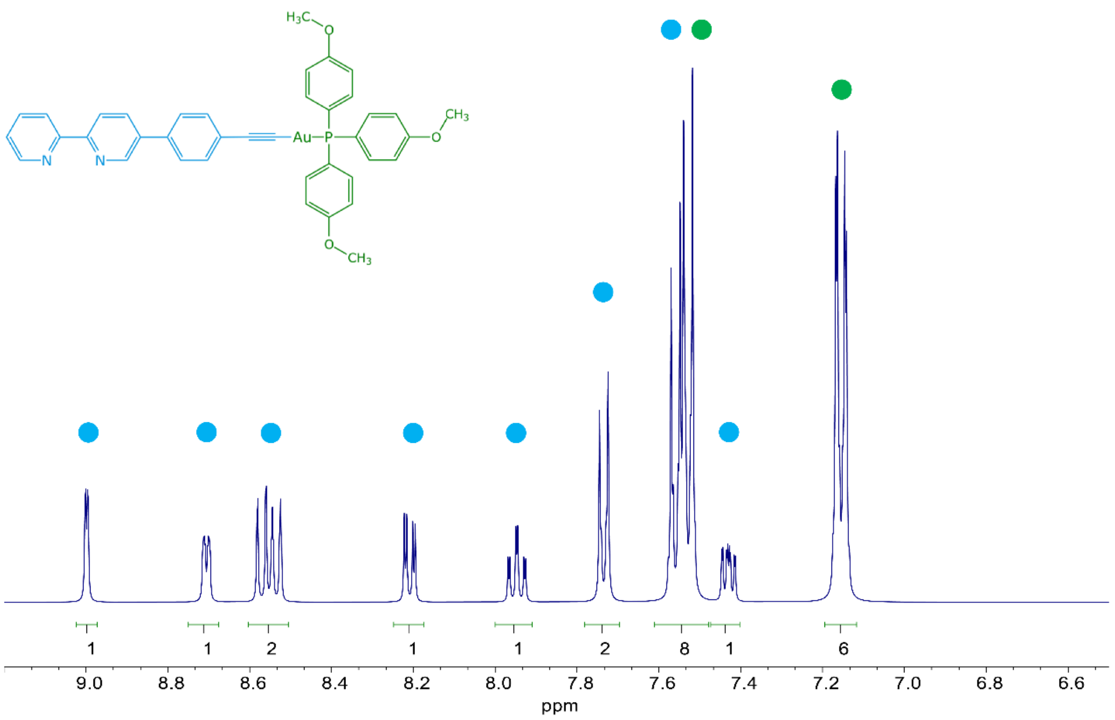

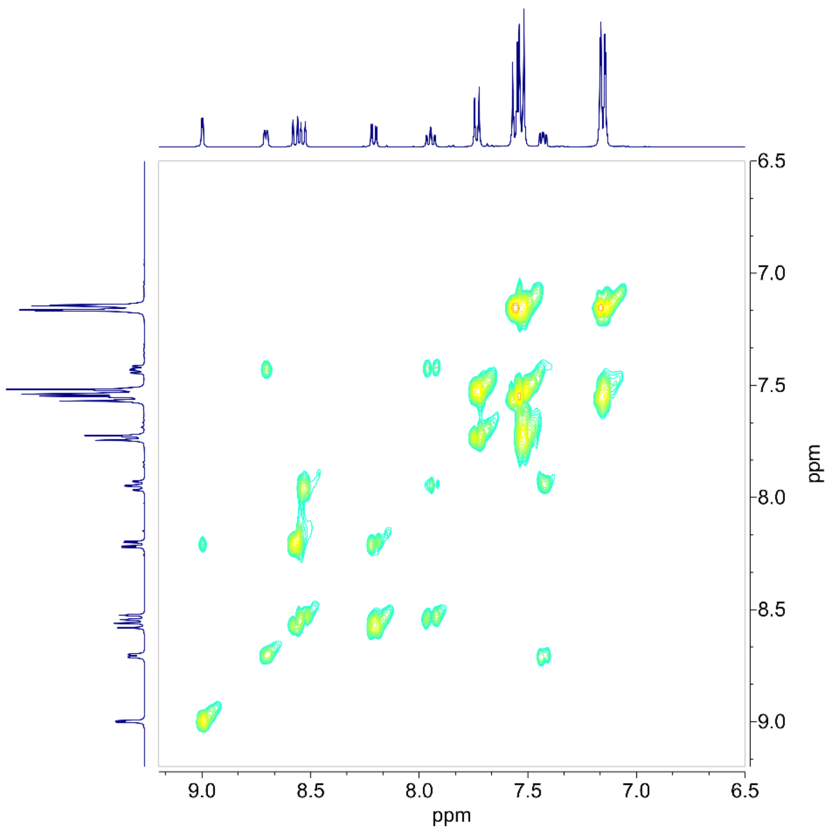

Figure S11. ^1H (top) and $^1H^1H$ COSY (bottom) spectra in aromatic range of Au(I) metalloligand.

1, acetone-d$_6$, 298 K

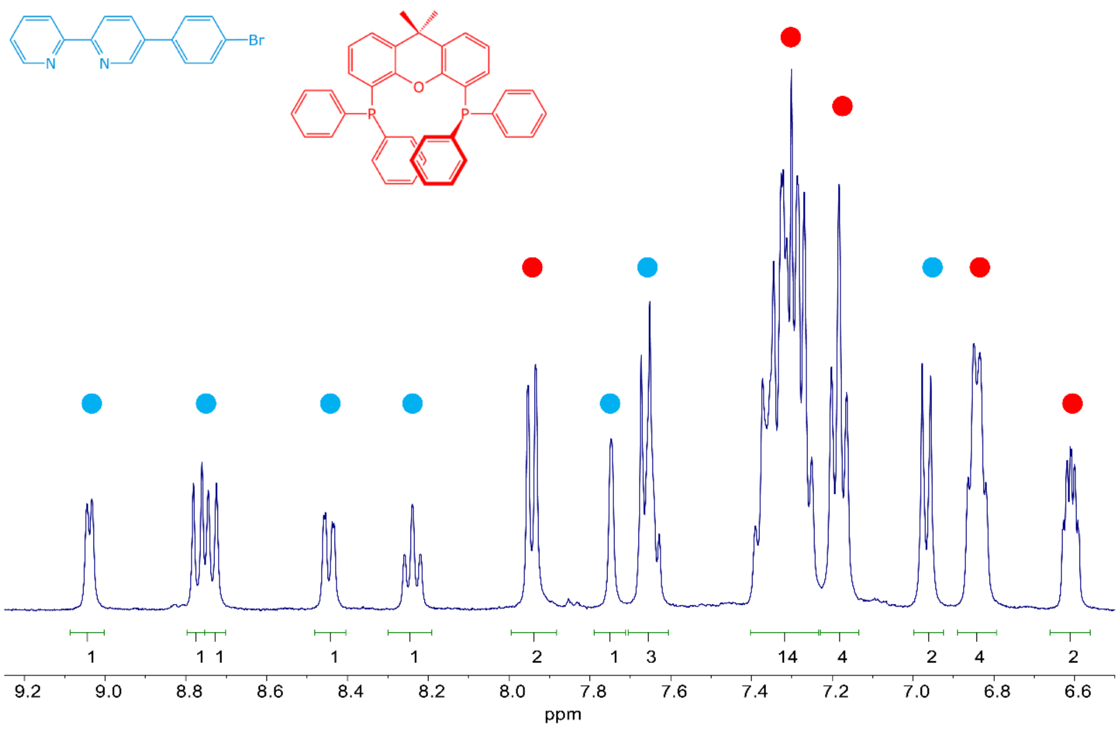

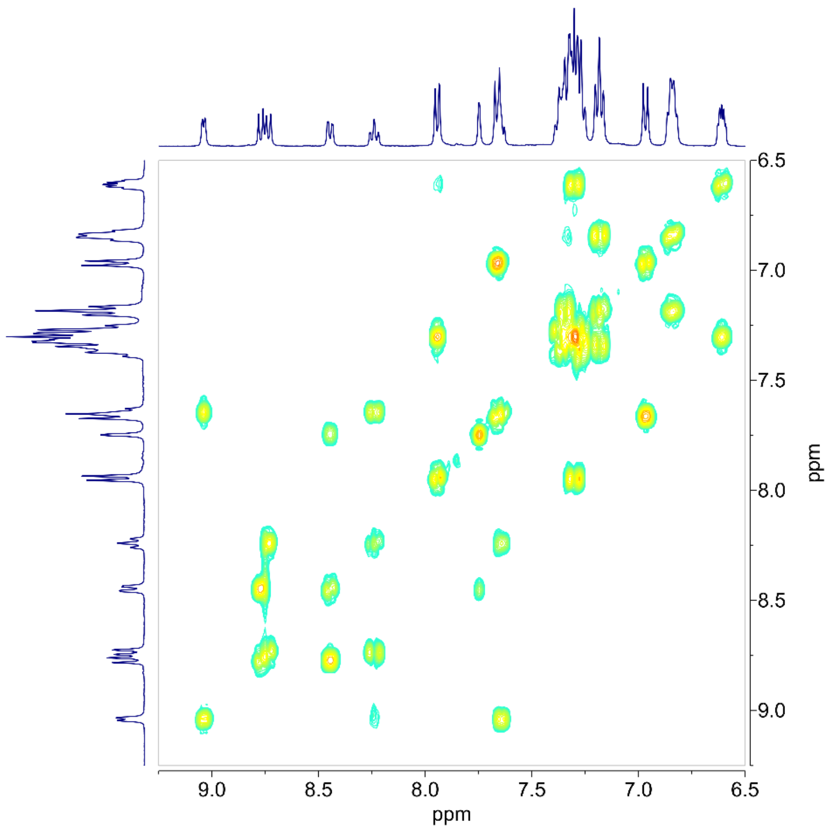

Figure S12. ^{1}H (top) and ^{1}H^{1}H COSY (bottom) NMR spectra of **1**, aromatic range.

2, acetone-d$_6$, 298 K

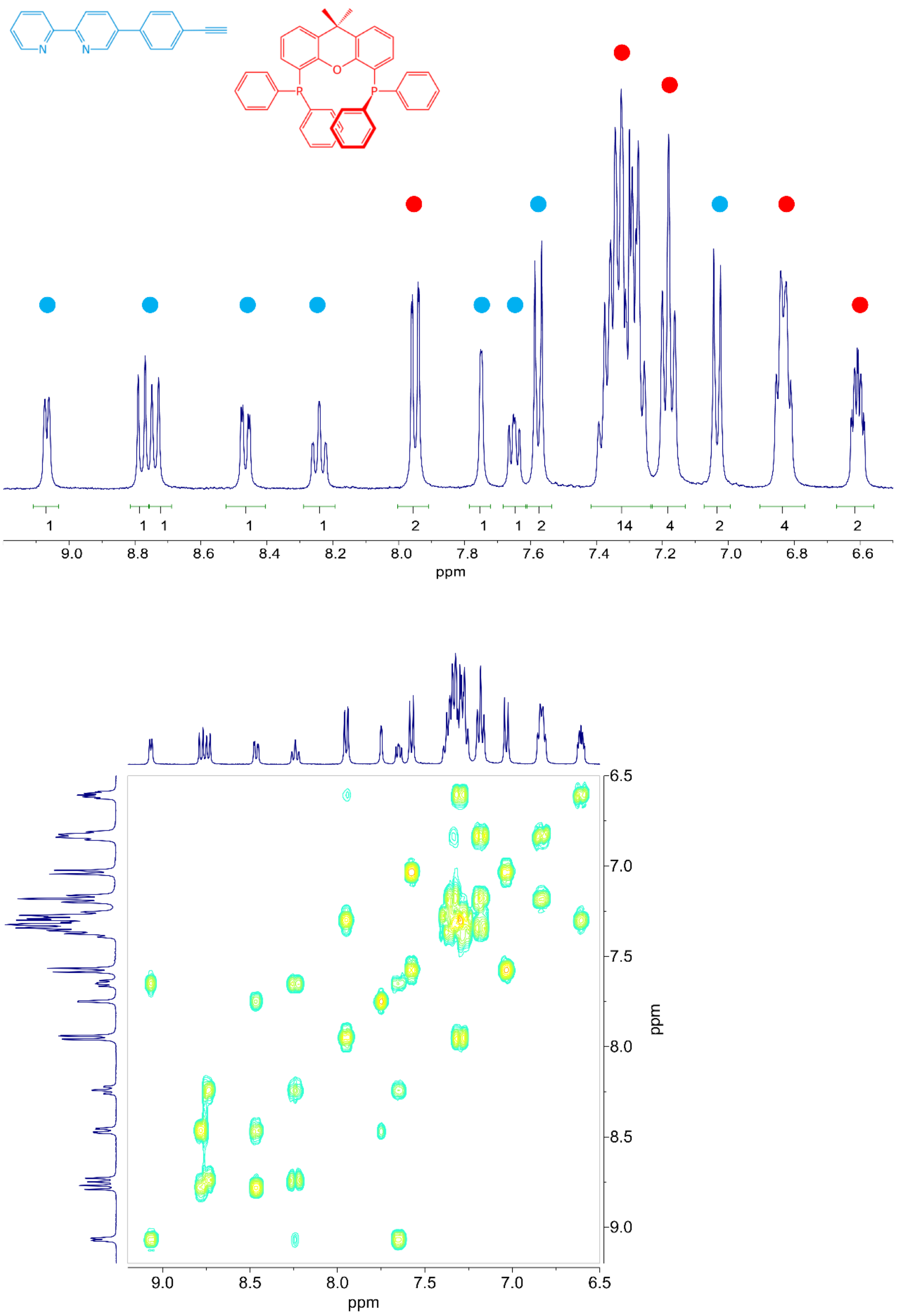

Figure S13. ^{1}H (top) and ^{1}H^{1}H COSY (bottom) NMR spectra of **2**, aromatic range.

3, acetone-d$_6$, 298 K

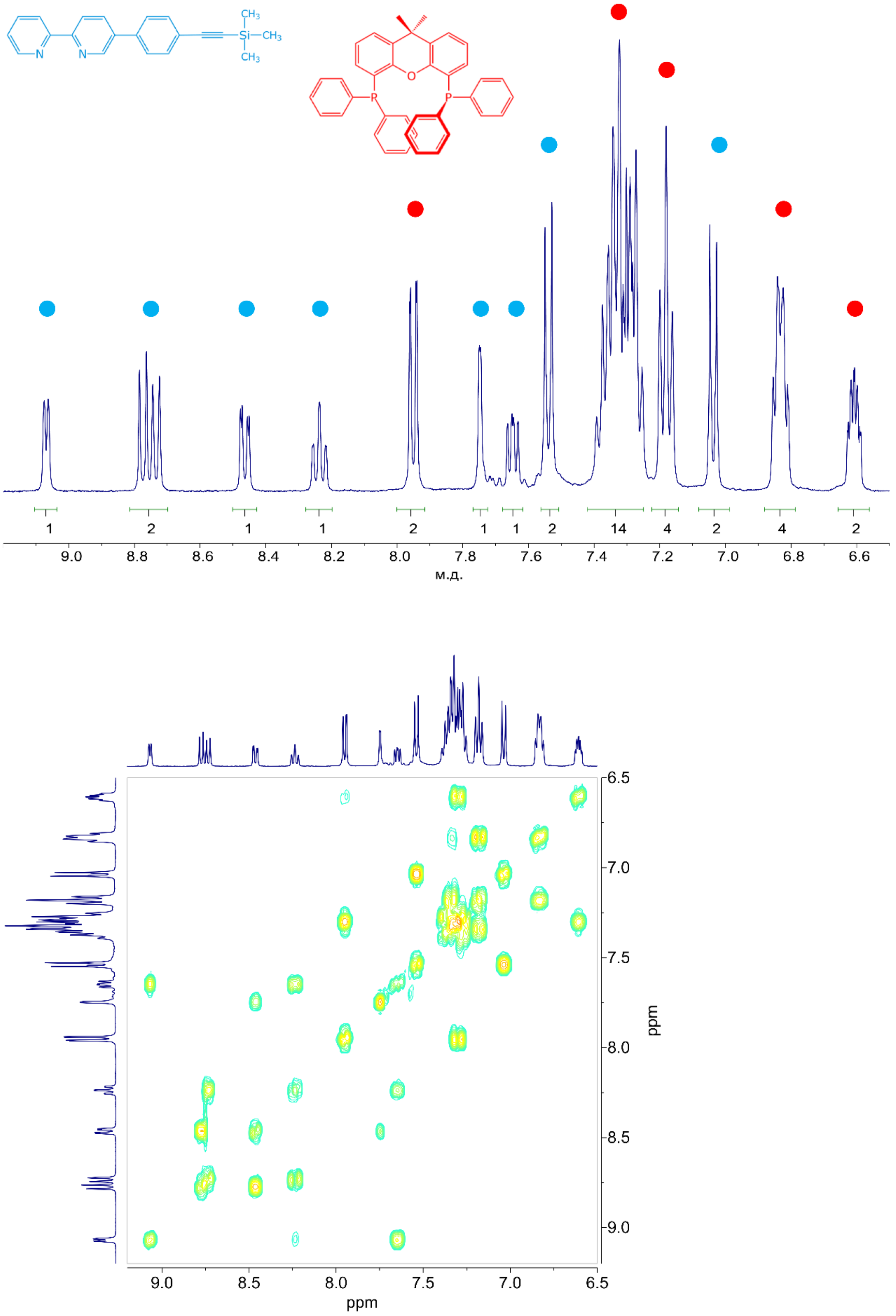

Figure S14. ^{1}H (top) and ^{1}H^{1}H COSY (bottom) NMR spectra of **3**, aromatic range.

4, acetone-d$_6$, 298 K

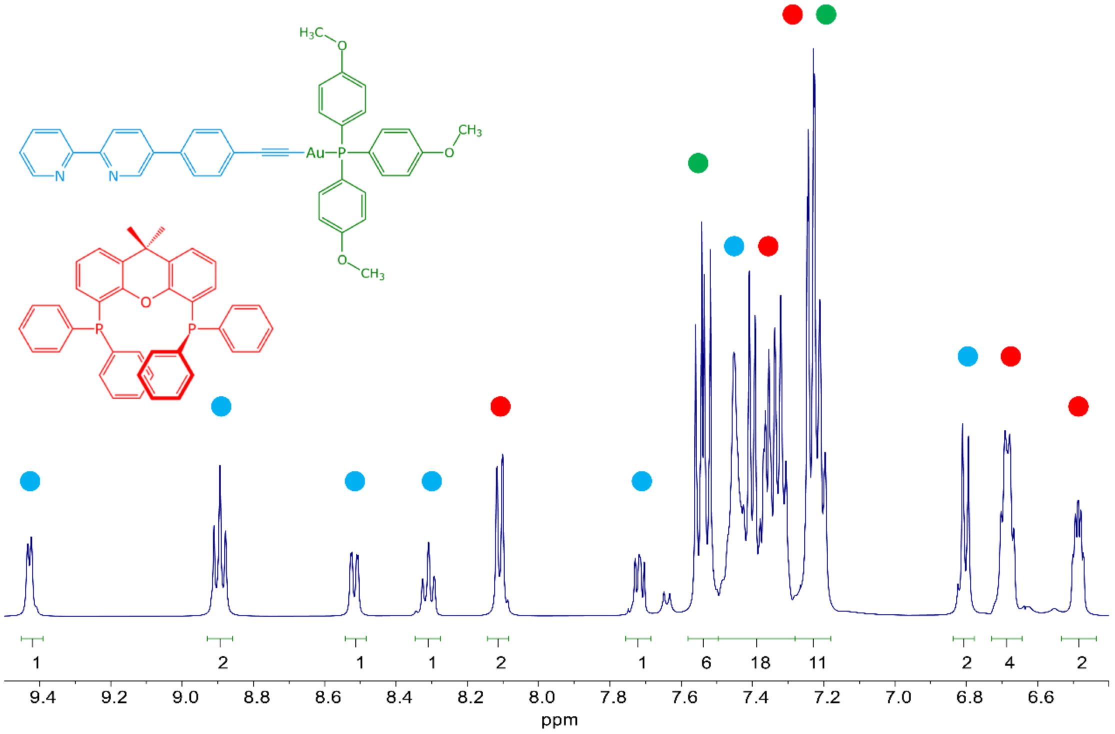

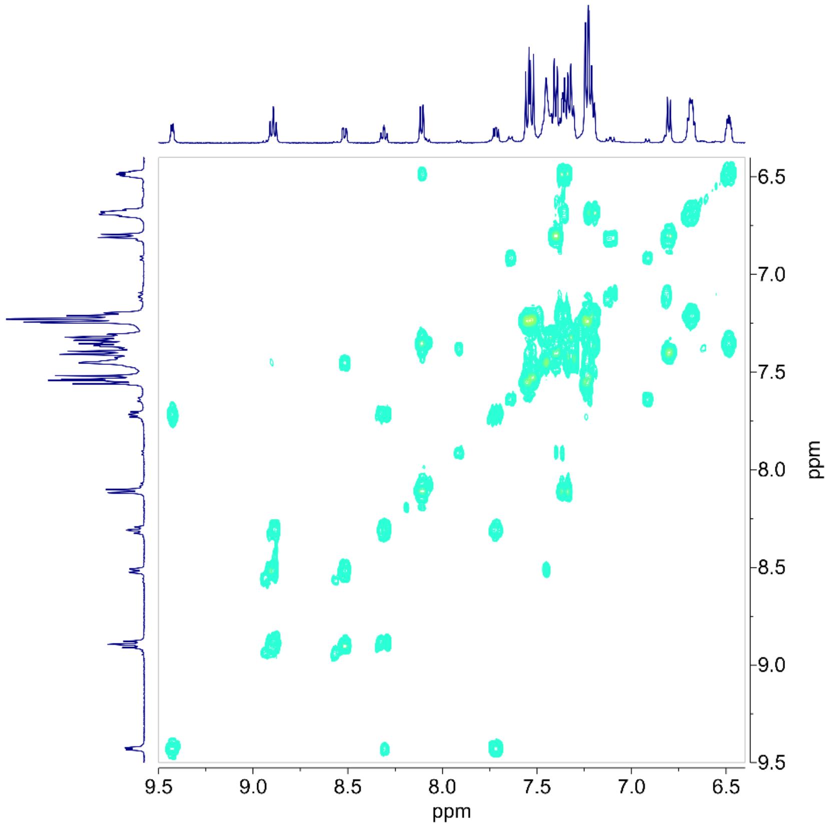

Figure S15. ^{1}H (top) and ^{1}H^{1}H COSY (bottom) NMR spectra of **4**, aromatic range.

5, acetone-d$_6$, 298 K

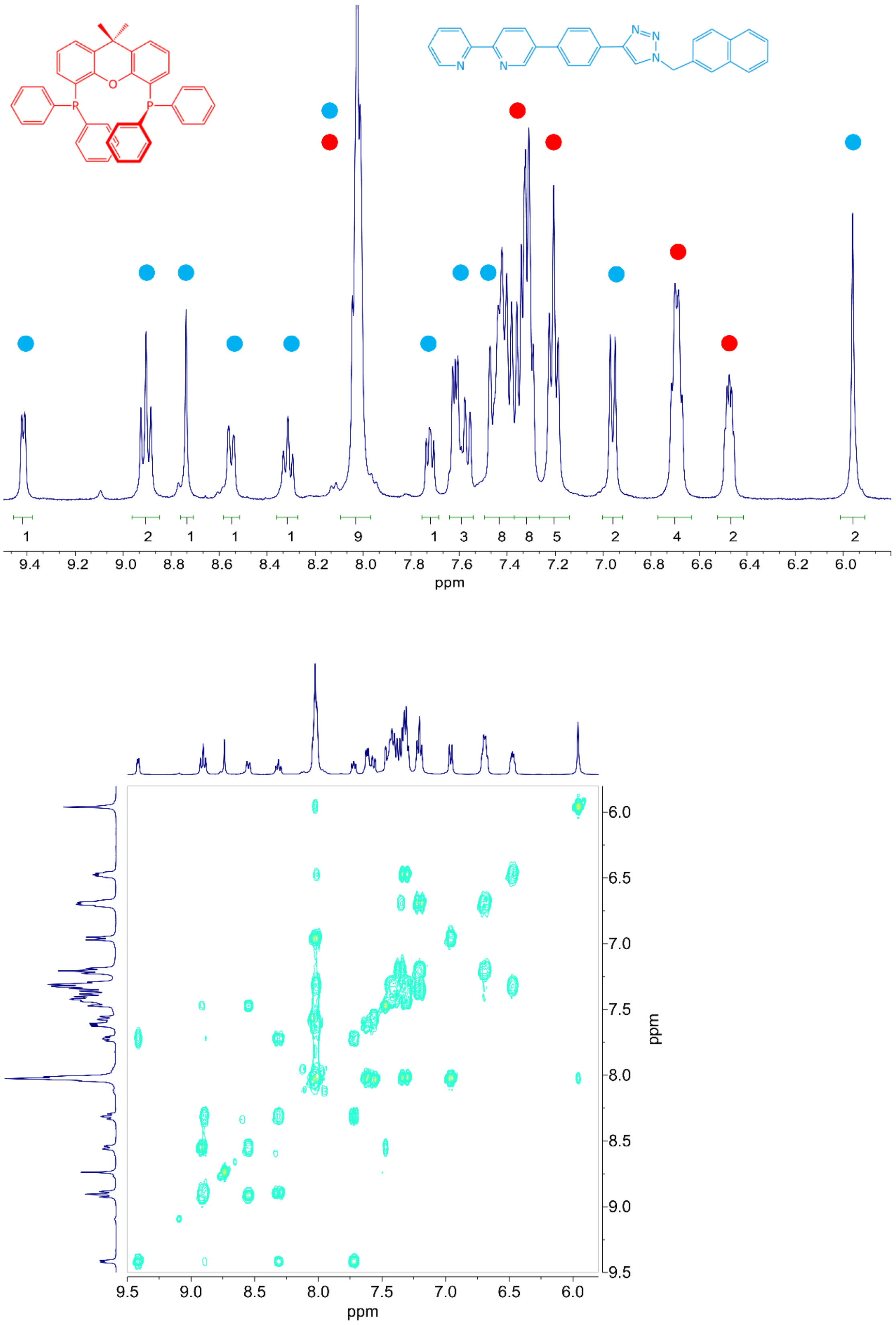

Figure S16. ^{1}H (top) and ^{1}H^{1}H COSY (bottom) NMR spectra of **5**, aromatic range.

6, acetone-d$_6$, 233 K

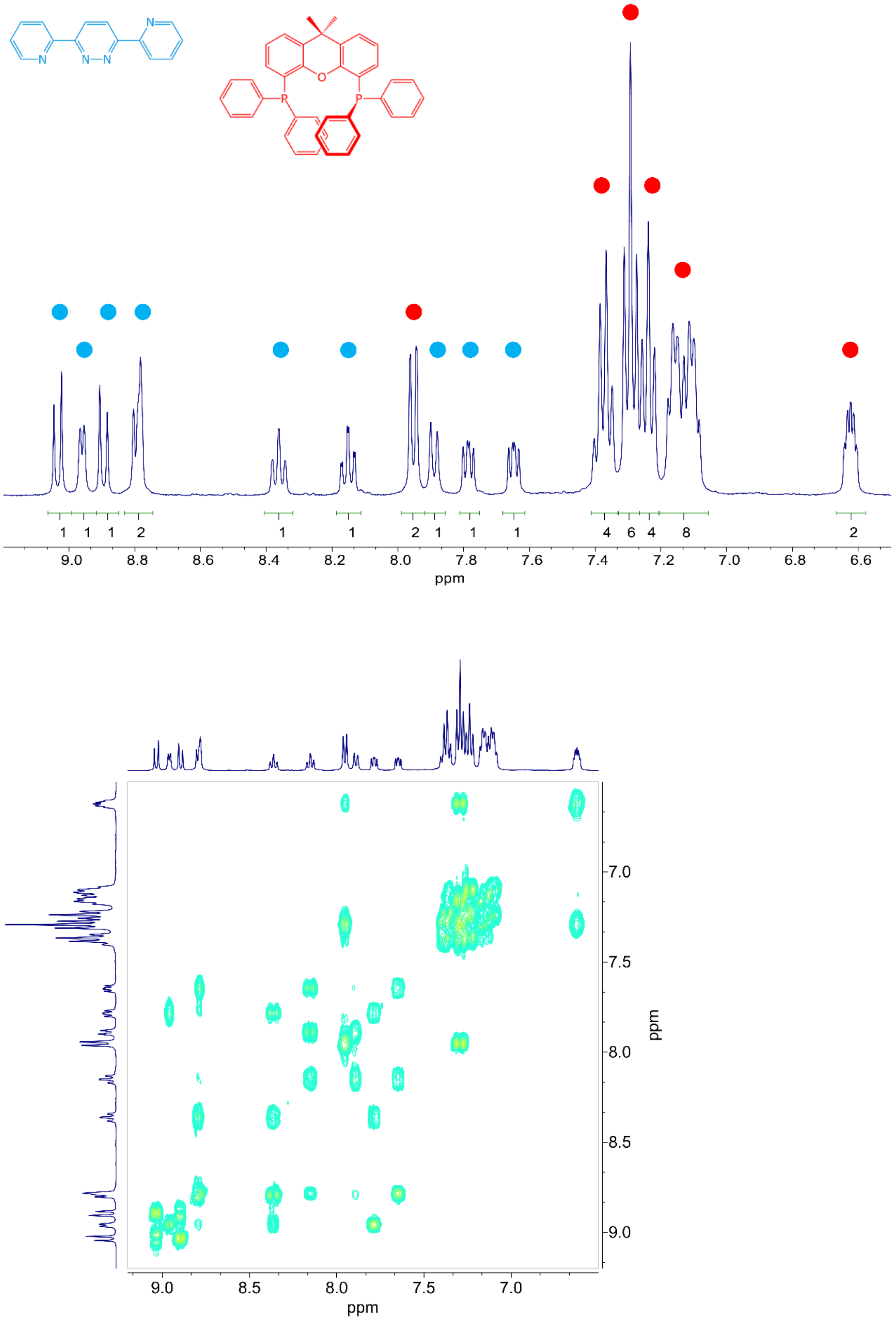

Figure S17. ^{1}H (top) and $^{1}H^{1}H$ COSY (bottom) NMR spectra of **6**, aromatic range.

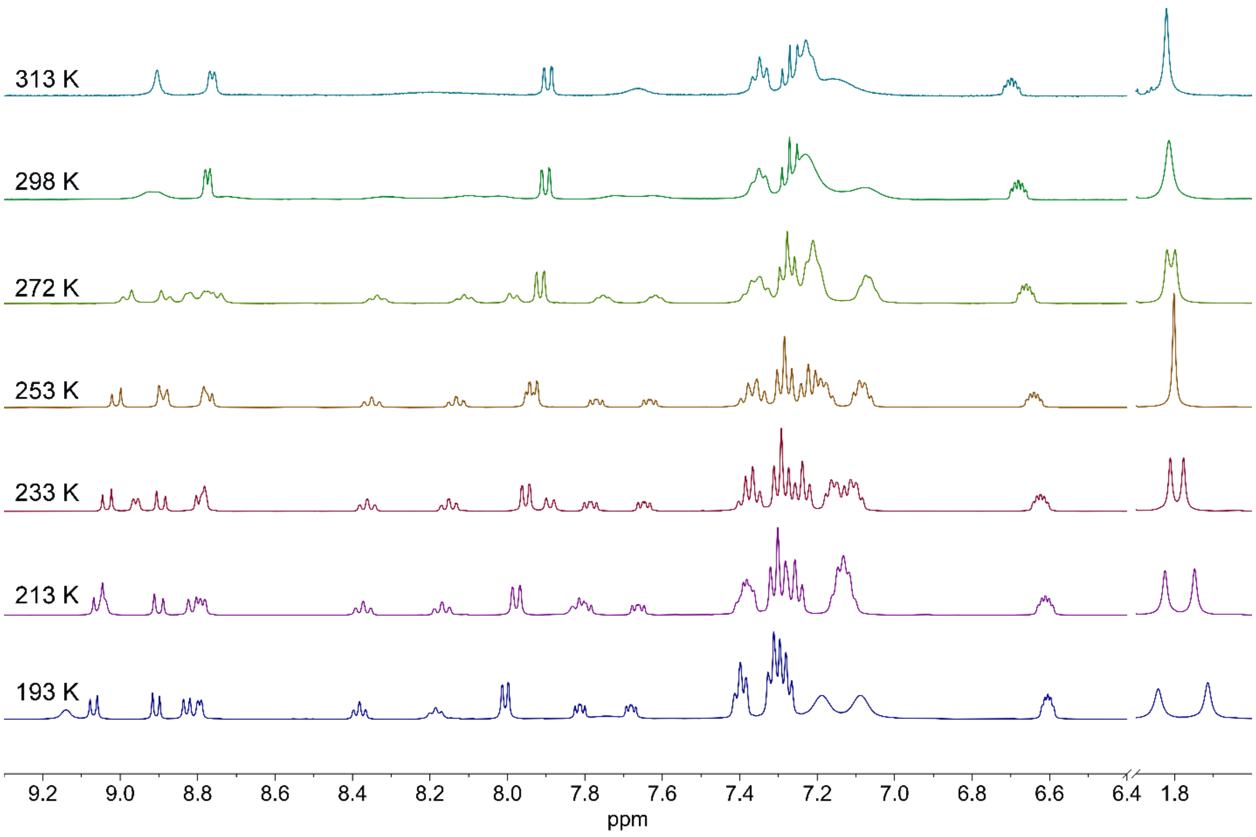

Figure S18. Variable temperature ^{1}H NMR spectra of **6**, acetone-d_6

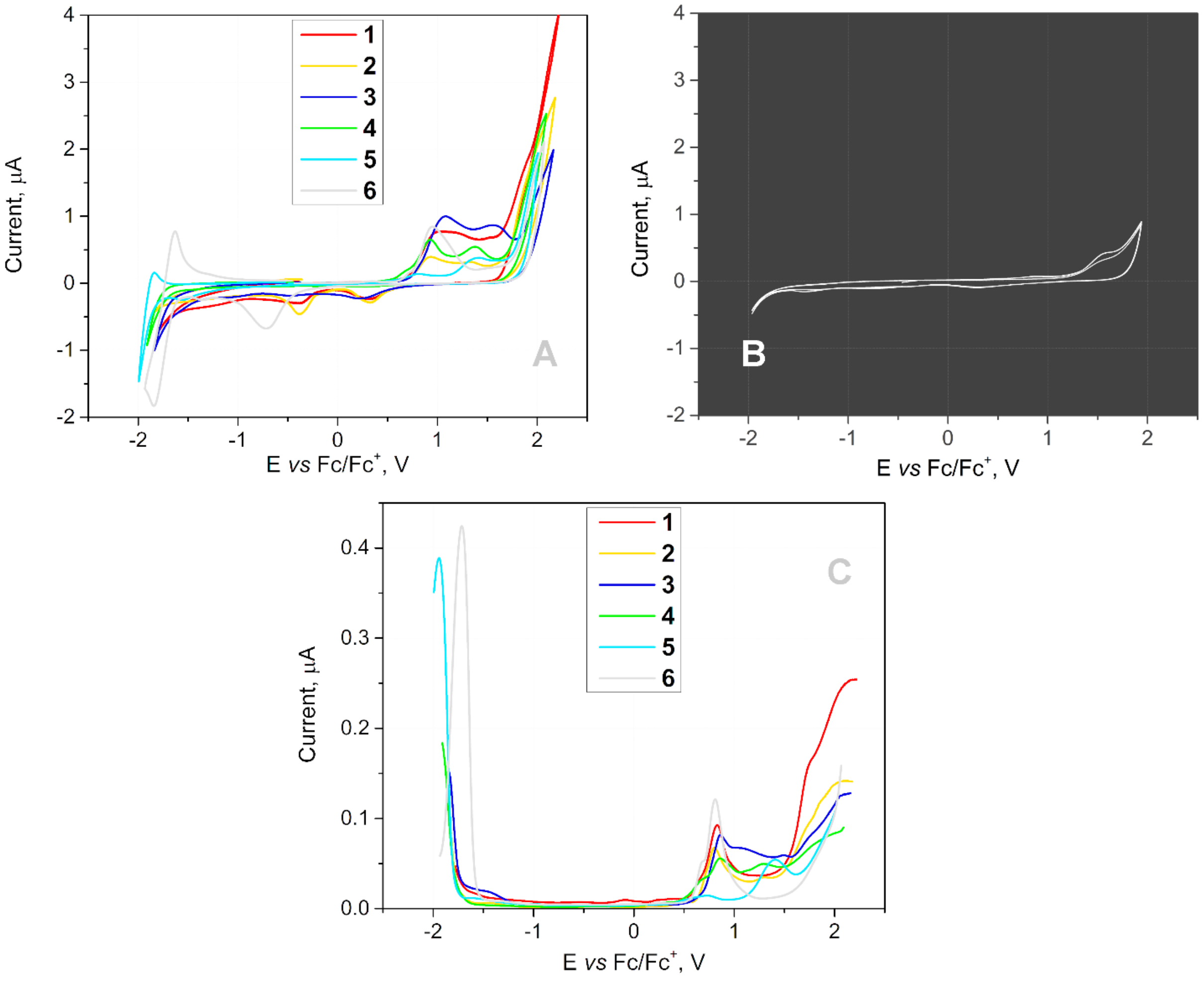

Figure S19. (A) Cyclic voltammograms of **1**–**6** in DCE solution with 0.1 M TBATFB as the supporting electrolyte referenced versus a Fc/Fc^{+} couple with a scan rate of 0.1 V s^{-1}; (B) background CV of DCE solution of 0.1 M TBATFB; (C) differential pulse voltammogram of **1**–**6**.

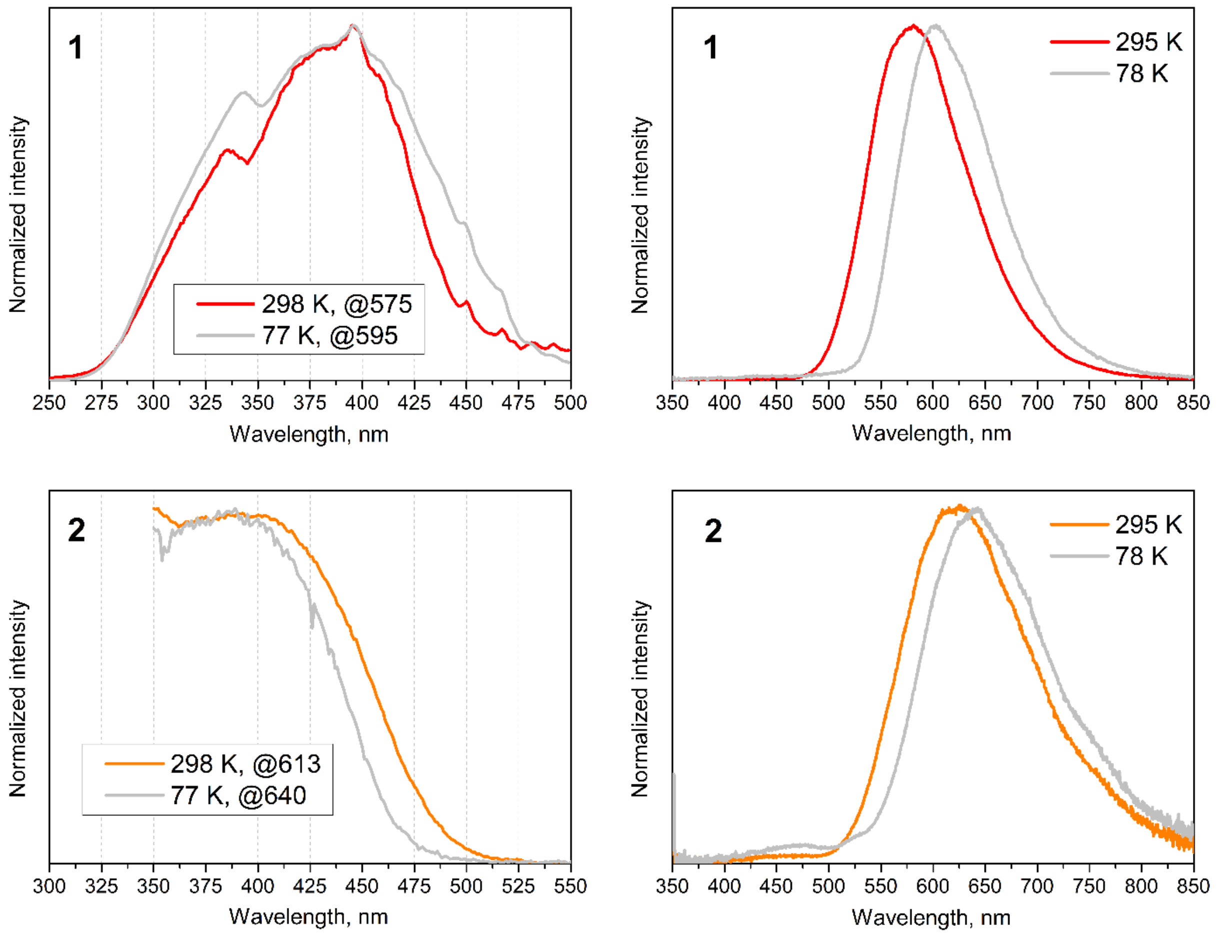

Figure S20. Normalized excitation (left) and emission (right, λ_{exct} = 351 nm) spectra of **1-2** in solid state at variable temperature.

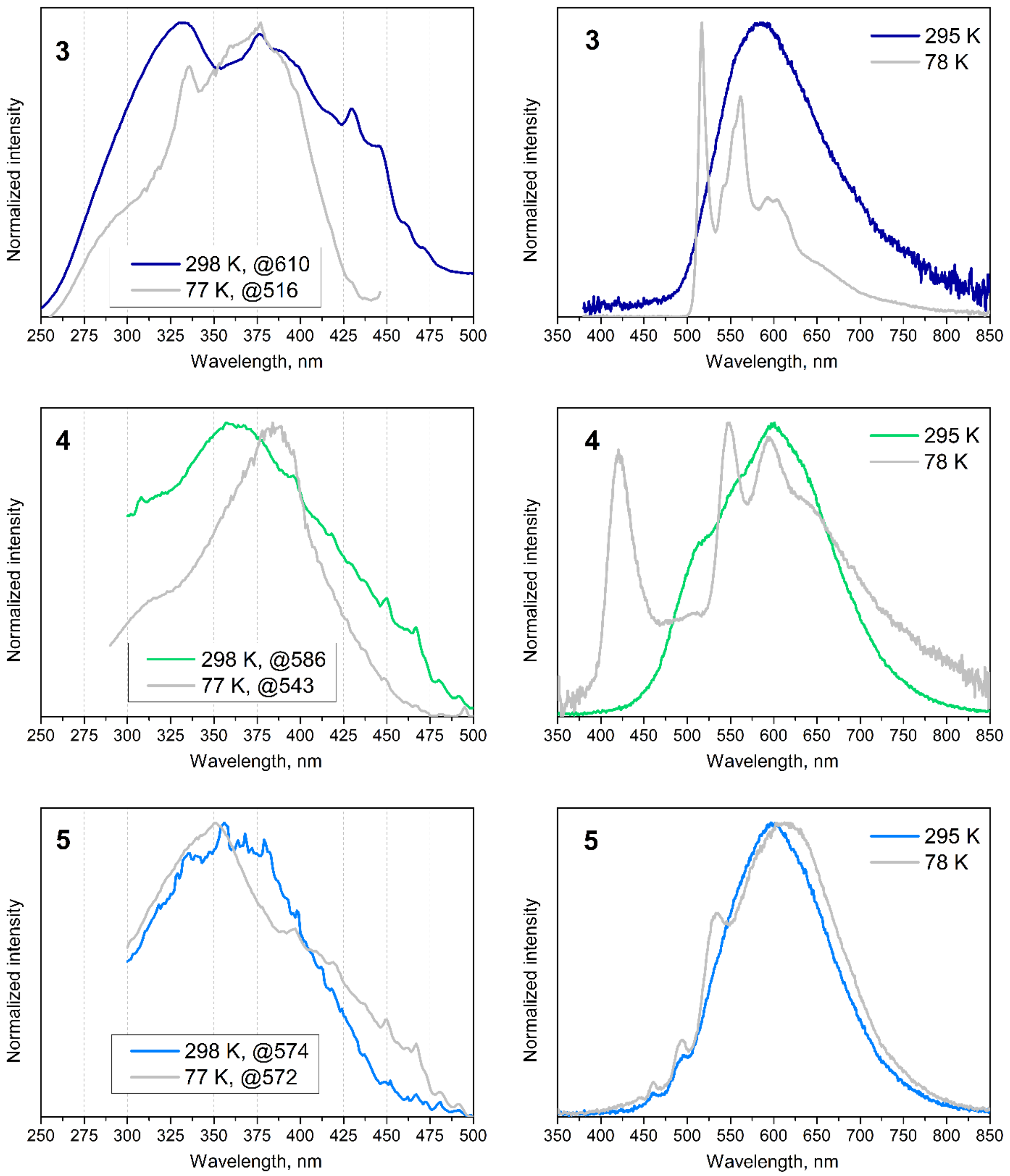

Figure S21. Normalized excitation (left) and emission (right, λ_{exct} = 351 nm) spectra of **3-5** in solid state at variable temperature.

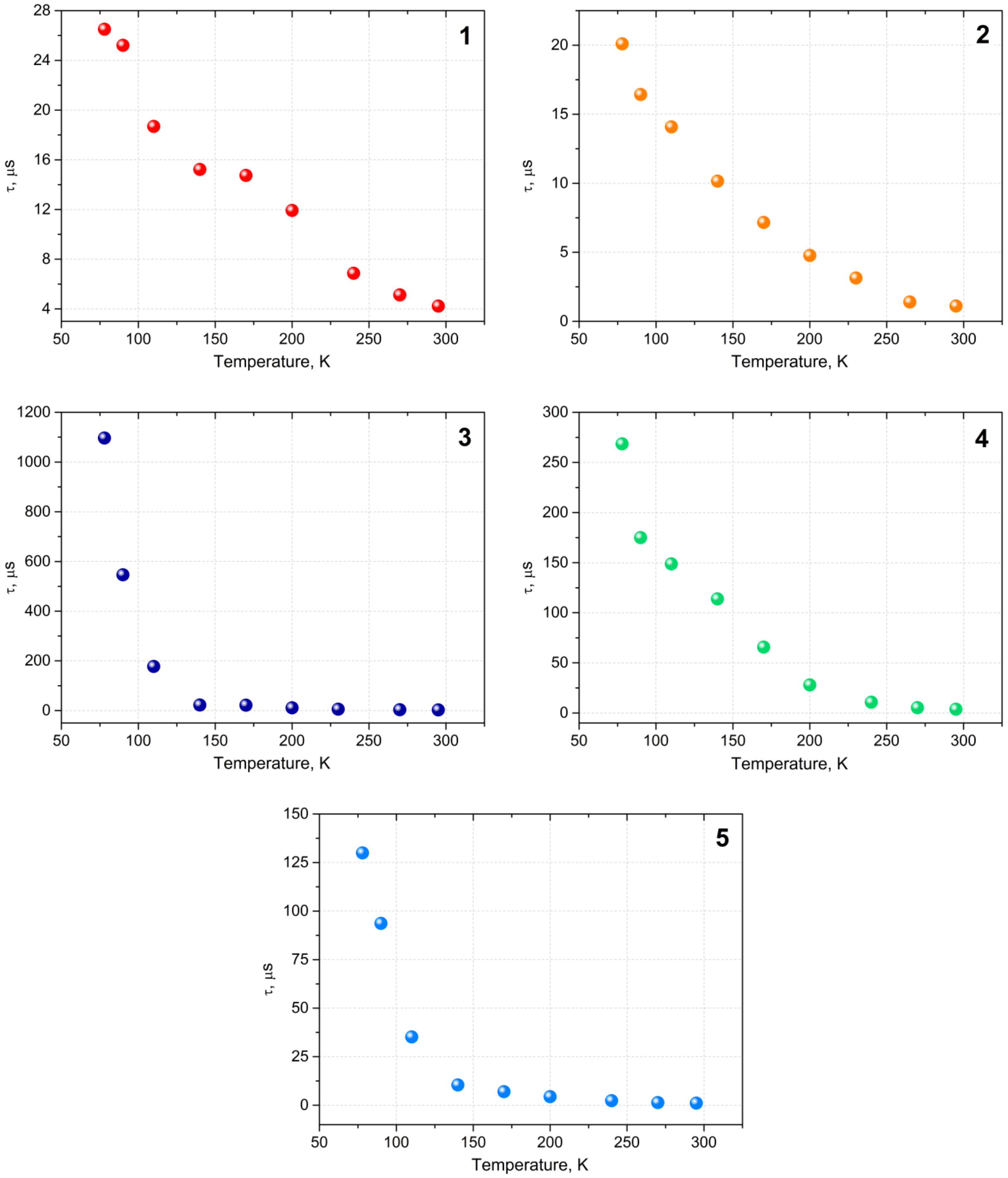

Figure S22. Average lifetime τ_{aver} at different temperatures of **1**–**5** in solid state, λ_{exct} = 351 nm.

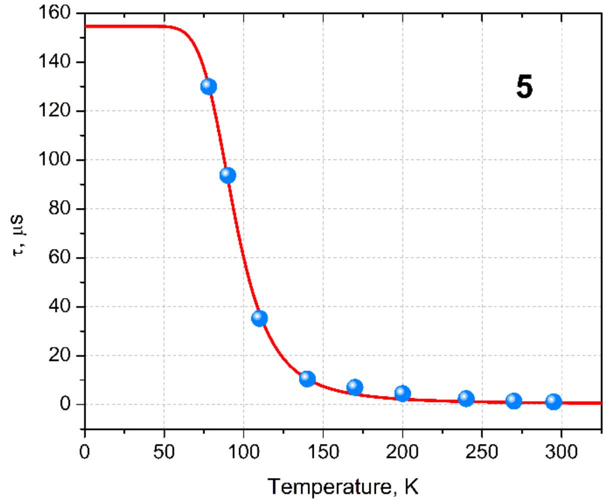

Figure S23. Temperature dependence and fitting curve of the lifetimes observed (τ_{aver}) for **5**, λ_{exct} = 351 nm.

The following equation is used for the fitting [18]

$$\tau(T) = \frac{3 + \exp(-\frac{\Delta E(S_1 - T_1)}{k_B T})}{\frac{3}{\tau(T_1)} + \frac{1}{\tau(S_1)} + \exp(-\frac{\Delta E(S_1 - T_1)}{k_B T})}$$

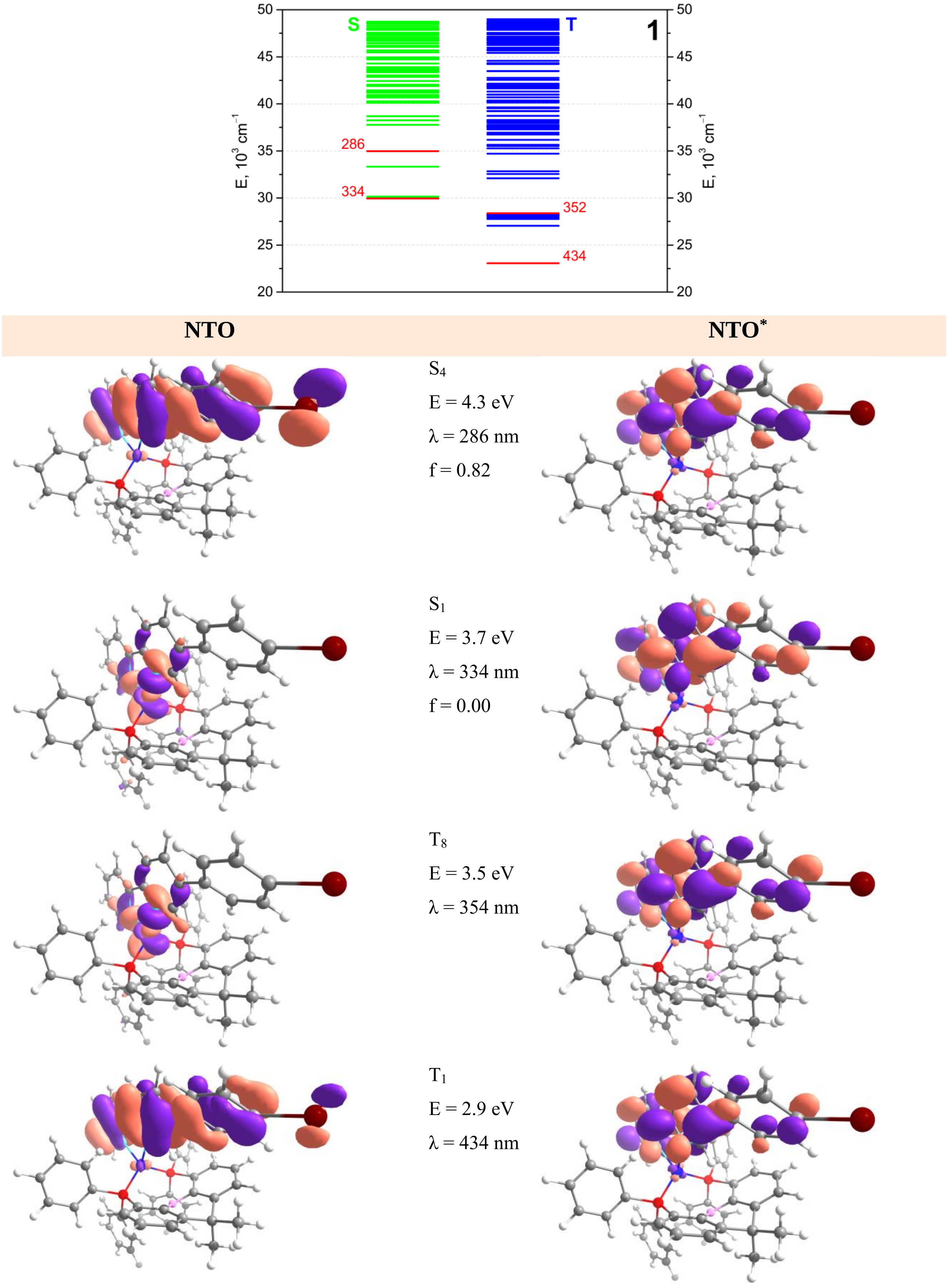

Figure S24. Energy level diagram and natural transition orbitals (NTOs) for the most important low-lying excited states in **1** as obtained from TDDFT calculations.

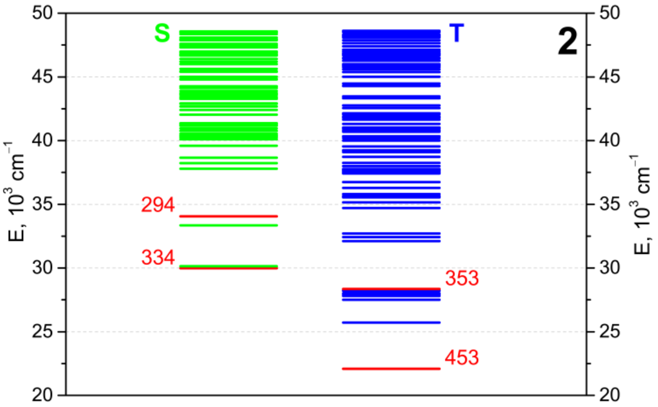

NTO		NTO*
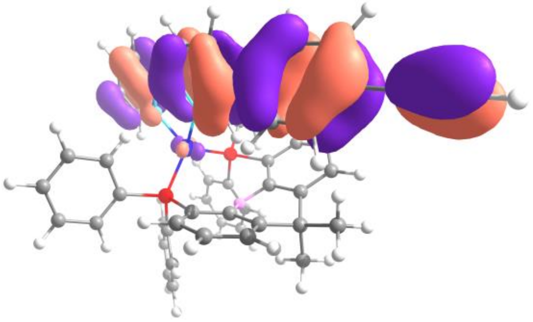	S_4<br>E = 4.2 eV<br>λ = 294 nm<br>f = 1.01	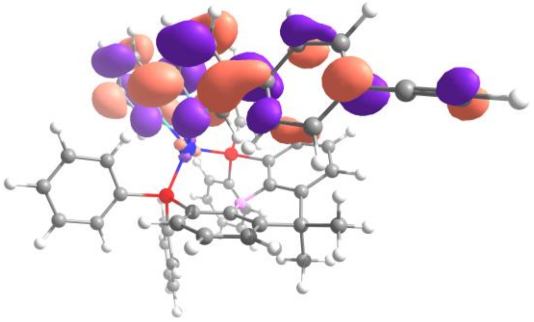
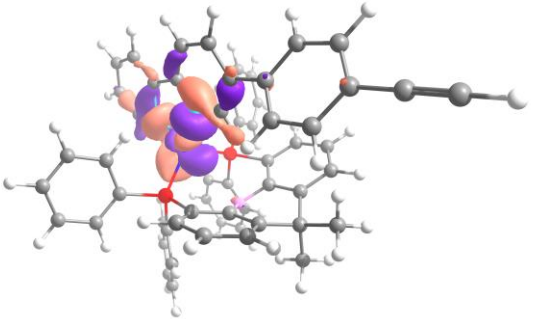	S_1<br>E = 3.7 eV<br>λ = 333 nm	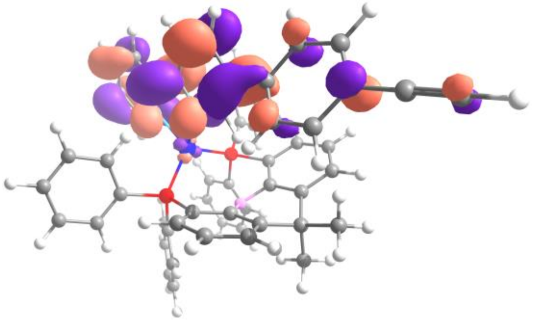
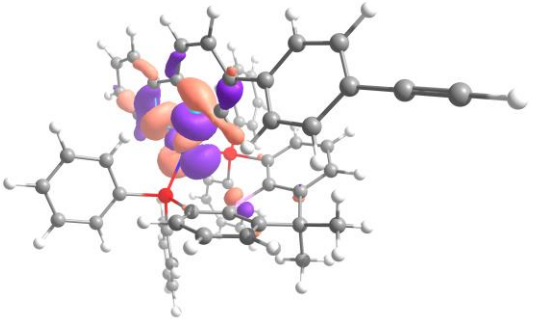	T_9<br>E = 3.5 eV<br>λ = 354 nm	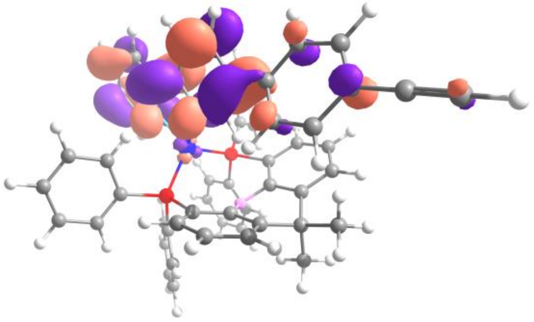
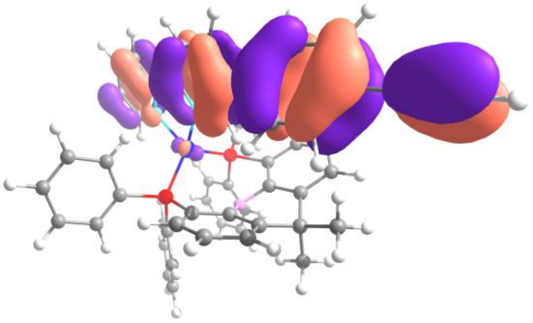	T_1<br>E = 2.7 eV<br>λ = 453 nm	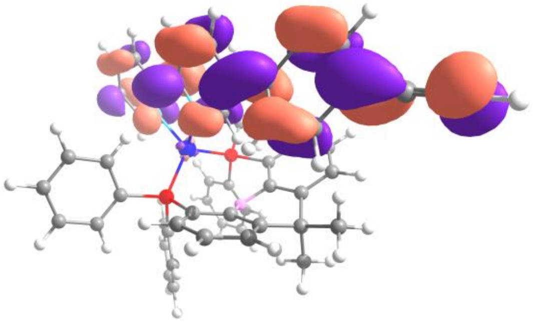

Figure S25. Energy level diagram and NTOs for the most important low-lying excited states in **2** as obtained from TDDFT calculations.

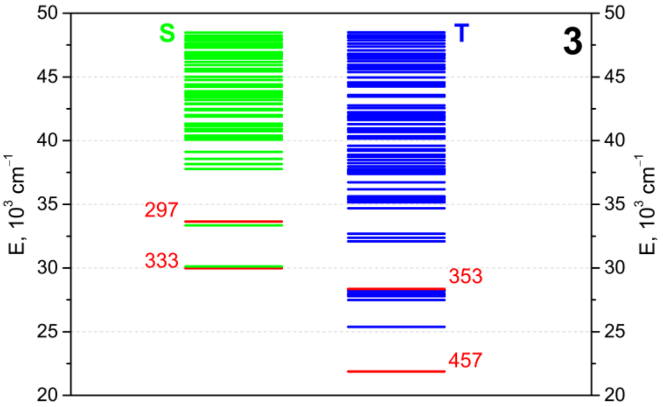

NTO	NTO*

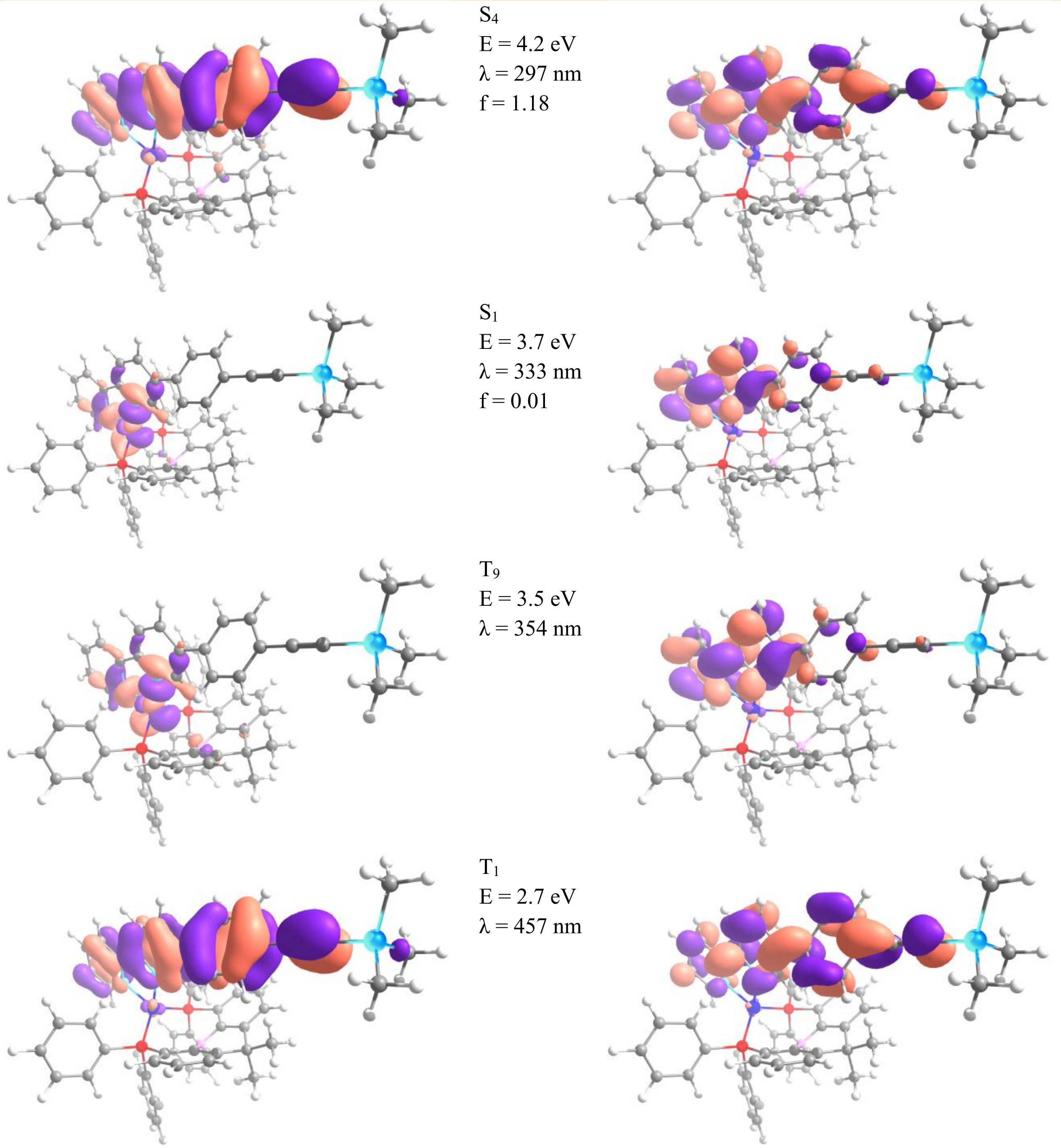

Figure S26. Energy level diagram and NTOs f for the most important low-lying excited states in **3** as obtained from TDDFT calculations.

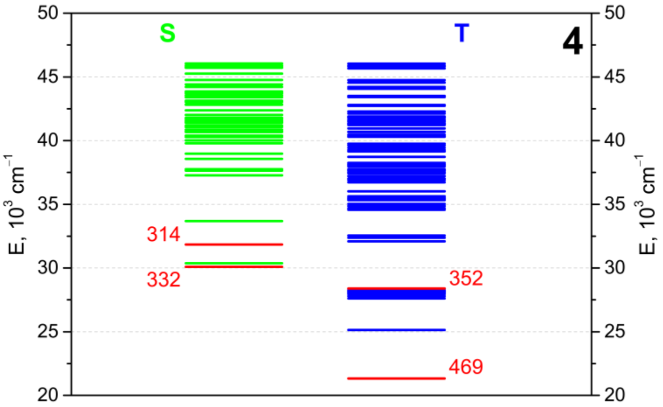

NTO	NTO*

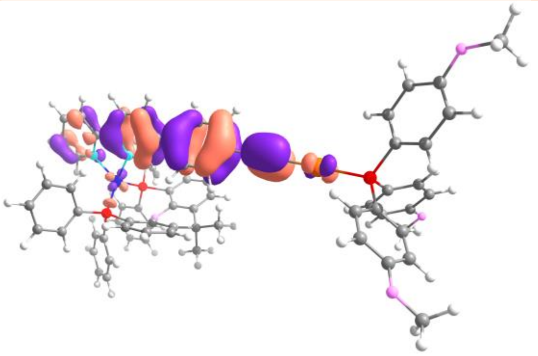

S_3
E = 3.9 eV
λ = 314 nm
f = 1.45

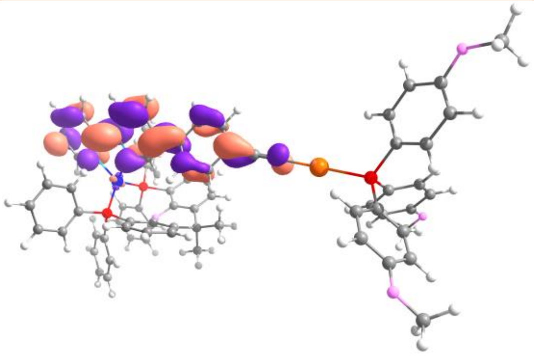

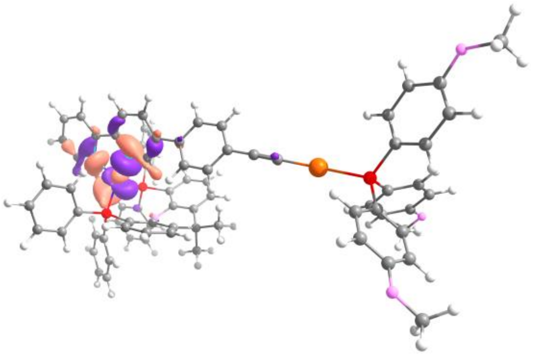

S_1
E = 3.7 eV
λ = 332 nm
f = 0.06

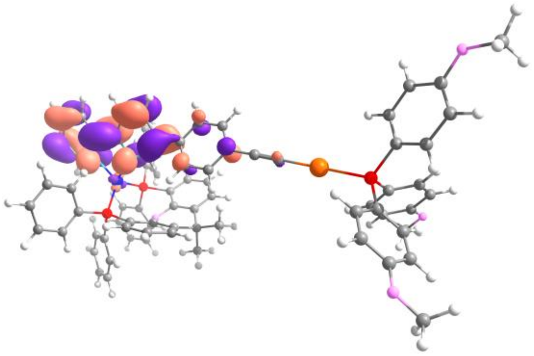

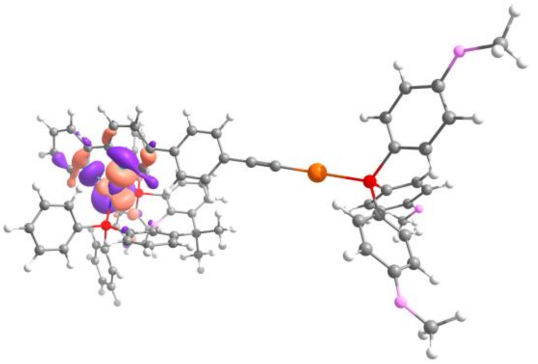

T_{13}
E = 3.5 eV
λ = 352 nm

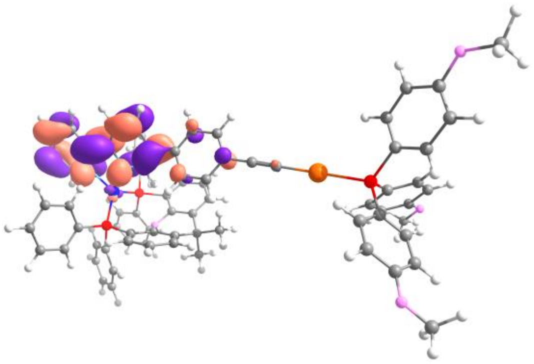

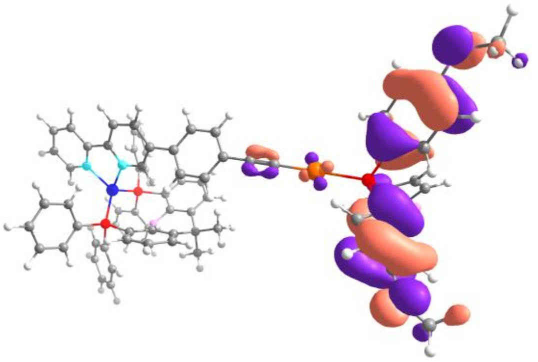

T_9
E = 3.5 eV
λ = 355 nm

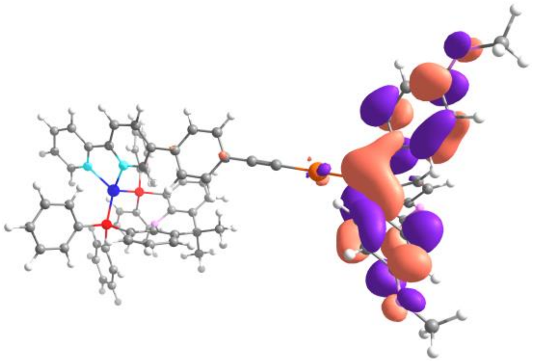

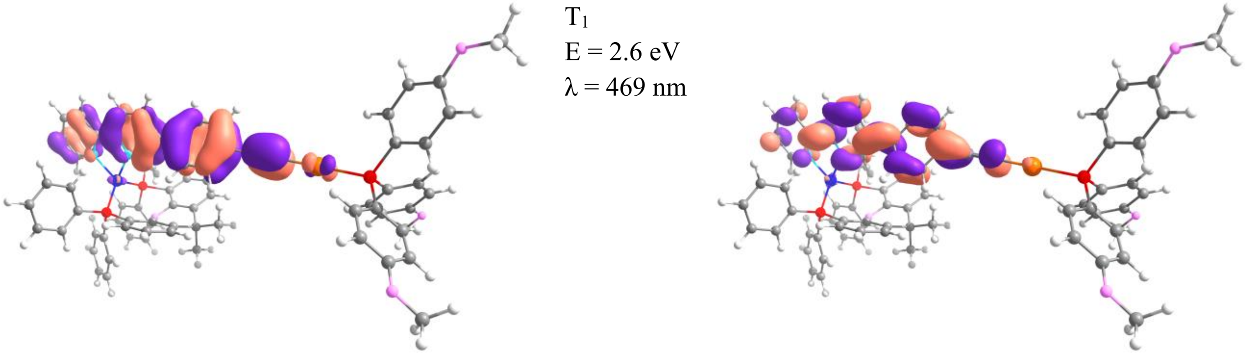

Figure S27. Energy level diagram and NTOs for the most important low-lying excited states in **4** as obtained from TDDFT calculations.

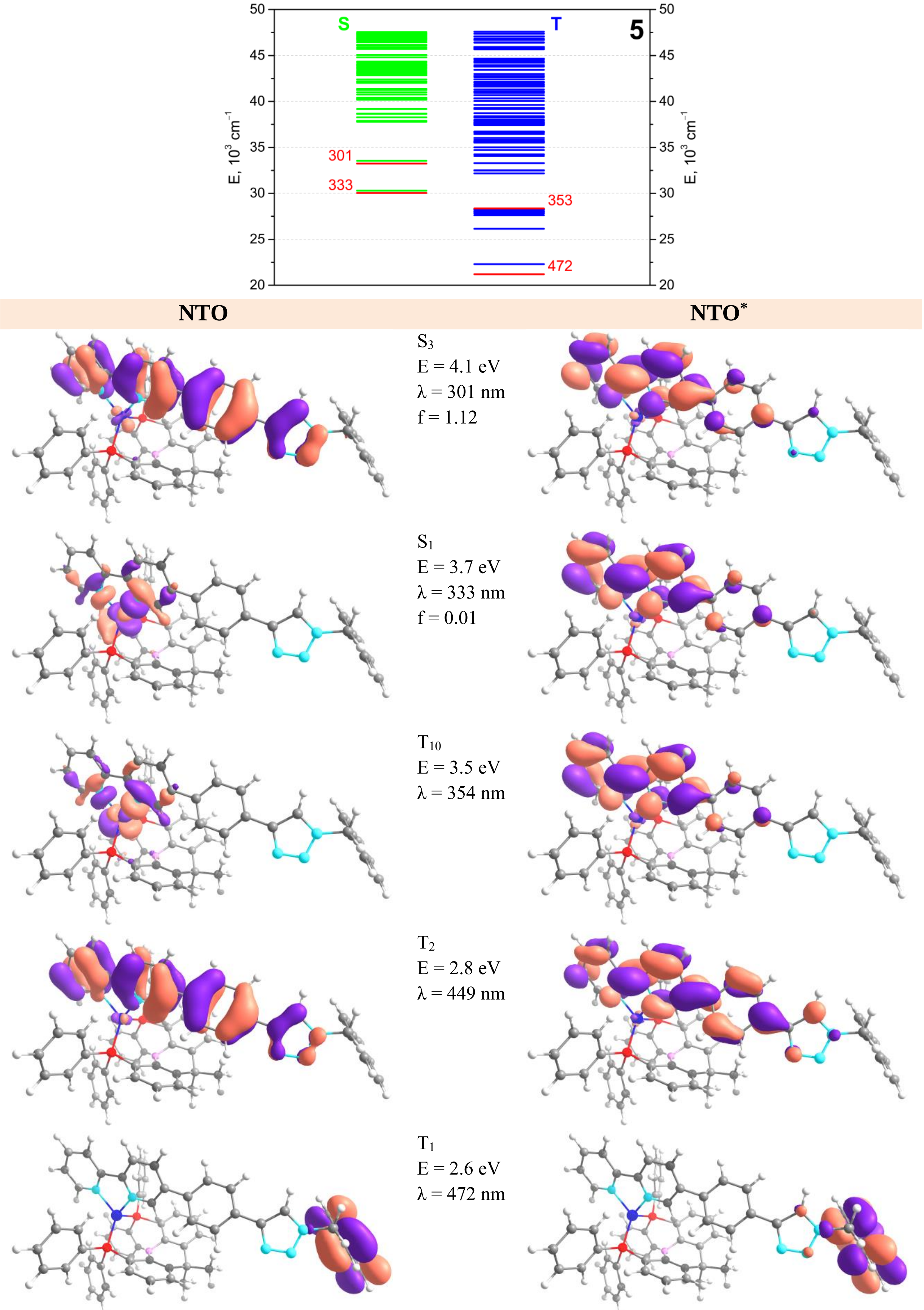

Figure S28. Energy level diagram and NTOs for the most important low-lying excited states in **5** as obtained from TDDFT calculations.

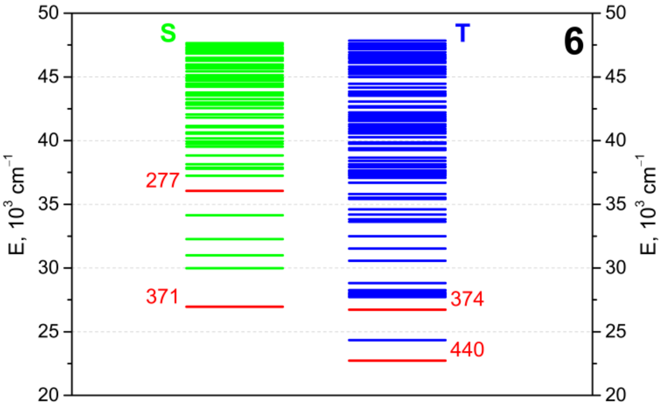

NTO		NTO*
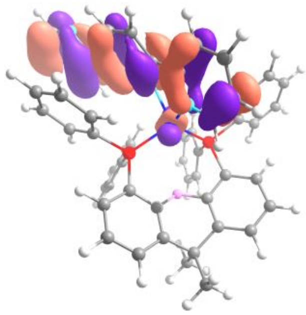	S_6<br>E = 4.5 eV<br>λ = 277 nm<br>f = 0.47	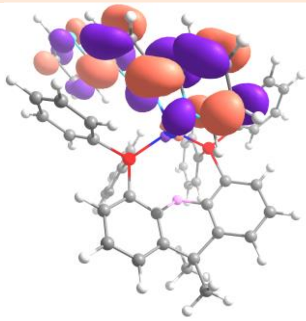
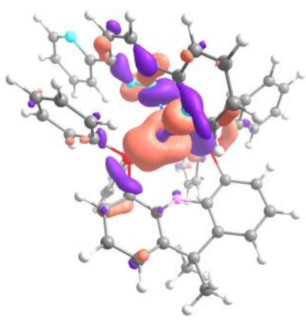	S_1<br>E = 3.3 eV<br>λ = 371 nm<br>f = 0.08	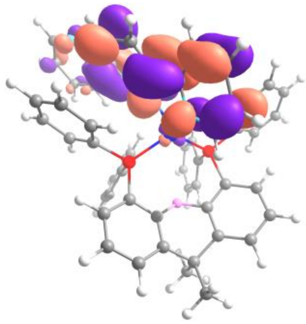
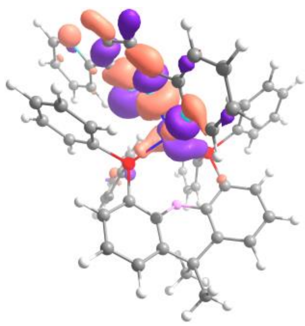	T_3<br>E = 3.3 eV<br>λ = 374 nm	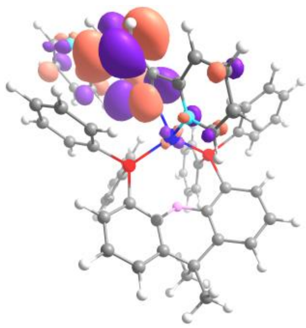
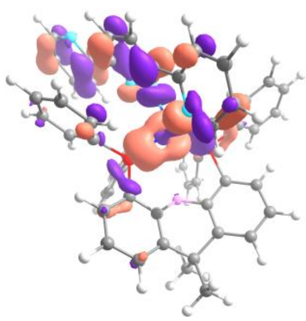	T_1<br>E = 2.8 eV<br>λ = 440 nm	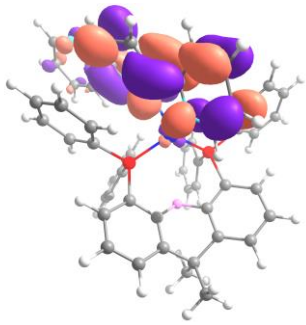

Figure S29. Energy level diagram and NTOs for the most important low-lying excited states in **6** as obtained from TDDFT calculations.

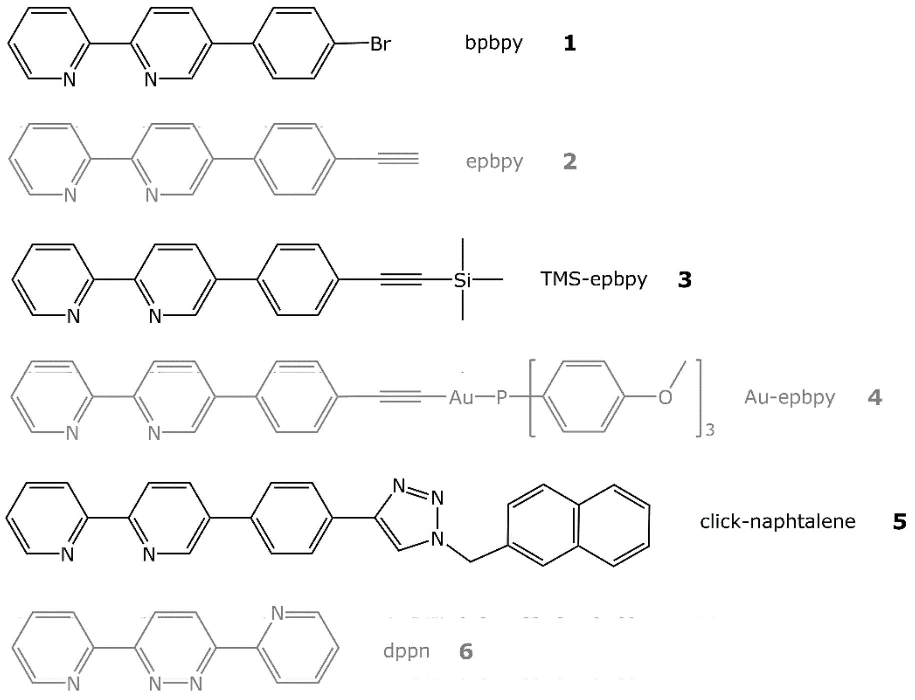

Figure S30. Abbreviations of N^N ligands correlated with compound **1**−**6** numeration.

References

1. *CrysAlisPro, Rigaku Oxford Diffraction*; Agilent Technologies: Version 1.171.39.35a, 2017;
2. Sheldrick, G.M. Crystal structure refinement with SHELXL. *Acta Crystallogr. Sect. C* **2015**, *71*, 3–8, doi:10.1107/S2053229614024218.
3. Sheldrick, G.M. SHELXT - Integrated space-group and crystal-structure determination. *Acta Crystallogr. Sect. A* **2015**, *71*, 3–8, doi:10.1107/S2053273314026370.
4. Dolomanov, O. V.; Bourhis, L.J.; Gildea, R.J.; Howard, J.A.K.; Puschmann, H. OLEX2: A complete structure solution, refinement and analysis program. *J. Appl. Crystallogr.* **2009**, *42*, 339–341, doi:10.1107/S0021889808042726.
5. Spek, A.L. PLATON SQUEEZE: a tool for the calculation of the disordered solvent contribution to the calculated structure factors. *Acta Crystallogr. Sect. C* **2015**, *71*, 9–18, doi:10.1107/S2053229614024929.
6. Keller, S.; Alkan-Zambada, M.; Prescimone, A.; Constable, E.C.; Housecroft, C.E. Extended π-Systems in Diimine Ligands in [Cu(P^P)(N^N)][PF6] Complexes: From 2,2′-Bipyridine to 2-(Pyridin-2-yl)Quinoline. *Crystals* **2020**, *10*, 255, doi:10.3390/cryst10040255.
7. Keller, S.; Pertegás, A.; Longo, G.; Martínez, L.; Cerdá, J.; Junquera-Hernández, J.M.; Prescimone, A.; Constable, E.C.; Housecroft, C.E.; Ortí, E.; et al. Shine bright or live long: substituent effects in [Cu(N^N)(P^P)]+ -based light-emitting electrochemical cells where N^N is a 6-substituted 2,2′-bipyridine. *J. Mater. Chem. C* **2016**, *4*, 3857–3871,

doi:10.1039/C5TC03725E.

8. Wang, W.-M.; Ju, P.; Jing, M.-H.; Yu, P.; Huang, Q. Synthesis and Characterization of Three New Emissive Mononuclear CuI Heteroleptic Complexes with Functionalized 6-Cyano-2,2′-bipyridine Chelating Ligands. *Aust. J. Chem.* **2020**, *73*, 640, doi:10.1071/CH19560.
9. Keller, S.; Prescimone, A.; Bolink, H.; Sessolo, M.; Longo, G.; Martínez-Sarti, L.; Junquera-Hernández, J.M.; Constable, E.C.; Ortí, E.; Housecroft, C.E. Luminescent copper(i) complexes with bisphosphane and halogen-substituted 2,2′-bipyridine ligands. *Dalt. Trans.* **2018**, *47*, 14263–14276, doi:10.1039/C8DT01338A.
10. Keller, S.; Prescimone, A.; La Placa, M.-G.; Junquera-Hernández, J.M.; Bolink, H.J.; Constable, E.C.; Sessolo, M.; Ortí, E.; Housecroft, C.E. The shiny side of copper: bringing copper(i) light-emitting electrochemical cells closer to application. *RSC Adv.* **2020**, *10*, 22631–22644, doi:10.1039/D0RA03824E.
11. Alkan-Zambada, M.; Keller, S.; Martínez-Sarti, L.; Prescimone, A.; Junquera-Hernández, J.M.; Constable, E.C.; Bolink, H.J.; Sessolo, M.; Ortí, E.; Housecroft, C.E. [Cu(P^P)(N^N)][PF6] compounds with bis(phosphane) and 6-alkoxy, 6-alkylthio, 6-phenyloxy and 6-phenylthio-substituted 2,2′-bipyridine ligands for light-emitting electrochemical cells. *J. Mater. Chem. C* **2018**, *6*, 8460–8471, doi:10.1039/C8TC02882F.
12. Weber, M.D.; Viciano-Chumillas, M.; Armentano, D.; Cano, J.; Costa, R.D. σ-Hammett parameter: a strategy to enhance both photo- and electro-luminescence features of heteroleptic copper(i) complexes. *Dalt. Trans.* **2017**, *46*, 6312–6323, doi:10.1039/C7DT00810D.
13. Keller, S.; Brunner, F.; Junquera-Hernández, J.M.; Pertegás, A.; La-Placa, M.-G.; Prescimone, A.; Constable, E.C.; Bolink, H.J.; Ortí, E.; Housecroft, C.E. CF 3 Substitution of [Cu(P^P)(bpy)][PF 6] Complexes: Effects on Photophysical Properties and Light-Emitting Electrochemical Cell Performance. *Chempluschem* **2018**, *83*, 217–229, doi:10.1002/cplu.201700501.
14. Brunner, F.; Babaei, A.; Pertegás, A.; Junquera-Hernández, J.M.; Prescimone, A.; Constable, E.C.; Bolink, H.J.; Sessolo, M.; Ortí, E.; Housecroft, C.E. Phosphane tuning in heteroleptic [Cu(N^N)(P^P)]+ complexes for light-emitting electrochemical cells. *Dalt. Trans.* **2019**, *48*, 446–460, doi:10.1039/C8DT03827A.
15. Addison, A.W.; Rao, T.N.; Reedijk, J.; van Rijn, J.; Verschoor, G.C. Synthesis, structure, and spectroscopic properties of copper(II) compounds containing nitrogen–sulphur donor ligands; the crystal and molecular structure of aqua[1,7-bis(N-methylbenzimidazol-2′-yl)-2,6-dithiaheptane]copper(II) perchlorate. *J. Chem. Soc., Dalt. Trans.* **1984**, 1349–1356, doi:10.1039/DT9840001349.
16. Yang, L.; Powell, D.R.; Houser, R.P. Structural variation in copper(I) complexes with

pyridylmethylamide ligands: structural analysis with a new four-coordinate geometry index, τ4. *Dalt. Trans.* **2007**, 955–964, doi:10.1039/B617136B.

17. Sillen, A.; Engelborghs, Y. The Correct Use of "Average" Fluorescence Parameters. *Photochem. Photobiol.* **1998**, *67*, 475–486, doi:10.1111/j.1751-1097.1998.tb09082.x.
18. Mahoro, G.U.; Fernandez-Cestau, J.; Renaud, J.; Coto, P.B.; Costa, R.D.; Gaillard, S. Recent Advances in Solid-State Lighting Devices Using Transition Metal Complexes Exhibiting Thermally Activated Delayed Fluorescent Emission Mechanism. *Adv. Opt. Mater.* **2020**, *8*, 2000260, doi:10.1002/adom.202000260.
